# Complex sphingolipid metabolism impacts cell division and plasmodesmal development in the moss *Physcomitrium patens*

**DOI:** 10.1093/plphys/kiaf549

**Published:** 2025-11-05

**Authors:** Linus Wegner, Cornelia Herrfurth, Ivo Feussner, Katrin Ehlers, Tegan M Haslam

**Affiliations:** Institute of Botany, Justus-Liebig University, Giessen 35392, Germany; Department of Plant Biochemistry, Albrecht-von-Haller-Institute for Plant Sciences, University of Goettingen, Goettingen 37077, Germany; Service Unit for Metabolomics and Lipidomics, Goettingen Center for Molecular Biosciences (GZMB), University of Goettingen, Goettingen 37077, Germany; Department of Plant Biochemistry, Albrecht-von-Haller-Institute for Plant Sciences, University of Goettingen, Goettingen 37077, Germany; Department of Plant Biochemistry, Goettingen Center for Molecular Biosciences (GZMB), University of Goettingen, Goettingen 37077, Germany; Institute of Botany, Justus-Liebig University, Giessen 35392, Germany; Department of Plant Biochemistry, Albrecht-von-Haller-Institute for Plant Sciences, University of Goettingen, Goettingen 37077, Germany

## Abstract

Developmental patterning and organ structure are elegantly simple in the moss *Physcomitrium patens*, which facilitates the cultivation and phenotypic characterization of severe mutant alleles. Essential membrane lipids, such as complex phosphosphingolipids (in plants, glycosyl inositol phosphorylceramides, GIPCs), are difficult to functionally characterize due to non-viable and pleiotropic phenotypes of mutants affected in their synthesis. Following the isolation and biochemical characterization of mutants affected in GIPC synthesis in *P. patens*, including *sphinganine-C4-hydroxylase/sphingoid base hydroxylase* (*s4h*/*sbh*) and *inositol phosphorylceramide synthase* (*ipcs*), we report some of their morphological, histological, and cytological phenotypes. We observed alterations in cell division, expansion, and differentiation. Specifically, the *s4h* knock-out mutant had abnormal cell division planes, as well as irregular depositions attached to cell walls. Severe *ipcs* mutant alleles showed frequent incomplete cell divisions, causing compromised cell autonomy as demonstrated by intercellular motility assays. These phenotypes suggest that sphingolipids impact both the orientation and proper formation of the cell plate during cytokinesis. Transmission electron microscopy revealed dramatic plasmodesmal structural defects in *ipcs* and *s4h* mutants, and these correlated with a macromolecule transport phenotype in *s4h*. Our methods can be used as a toolkit for quantifying growth, specifically cell division and plasmodesmal phenotypes in mosses, and our results illuminate key relationships between sphingolipid metabolism and fundamental cell functions. The severity of the observed defects in cell ultrastructure underscores both the resilience and the utility of *P. patens* as a model for investigating severe mutant phenotypes.

## Teaser

Sphingolipid deficiencies induced by targeted CRISPR mutagenesis produce extensive developmental defects in the moss *Physcomitrium patens*, impacting cytokinesis and plasmodesmal maturation.

## Introduction

**Figure 1.**
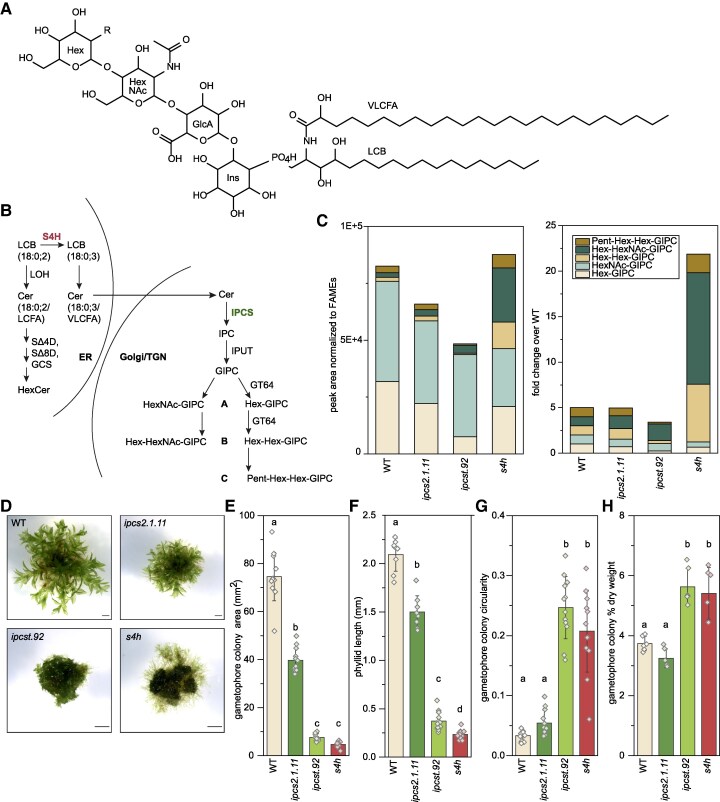
Altered sphingolipid content of mutant gametophores affects their overall growth. **A)** Chemical structure of a sphingolipid, specifically Hex-HexNAc-GIPC, 18:0;3/24:0;1, where 18:0;3 represents the long-chain base (LCB) with 18 carbons in length, no unsaturated bonds, and three hydroxylations, and 24:0;1 the very-long-chain fatty acid (VLCFA) moiety with 24 carbons in length, no unsaturated bonds, and a single hydroxylation. **B)** Simplified schematic of GIPC biosynthesis. Ceramide (Cer) assembly and backbone modifications occur on the endoplasmic reticulum, including SPHINGANINE-4-HYDROXYLASE (S4H, pink) hydroxylation of 18:0;2 LCBs to 18:0;3. In wild-type *P. patens*, only 18:0;3 LCBs are incorporated in GIPCs. Ceramides containing an 18:0;3 LCB and a VLCFA move into the Golgi and trans-Golgi network (TGN) where phospho-sugar headgroup additions build GIPCs. INOSITOL PHOSPHORYLCERAMIDE SYNTHASEs (IPCSs, green) catalyze the committed step in GIPC assembly. **C)** GIPC profiles of the mutants, based on ultra-high pressure liquid chromatography coupled with nanoelectrospray and tandem mass spectrometry (UPLC-nanoESI-MS/MS) quantification of lipids extracted from microsome enrichments from gametophores. Different GIPC class amounts are sums of individual MRM peak areas, normalized to the fatty acid content in each sample determined by FAMEs analysis (left). On the right, the same dataset, but processed as fold-change compared to the amount in wild type, where the amount of each GIPC type is set to a value of 1. The complete dataset is presented in Supplementary Fig. S1. **D)** Gametophores of the wild type, *ipcs2.1.11* single mutant, *ipcst.92* triple mutant, and *s4h* mutant, shown at different magnifications. All scale bars 1 mm. **E)** Gametophore colony area, **(F)** phyllid length, and **(G)** gametophore colony circularity after approximately two months of growth. **H)** % dry weight of gametophore tissue produced for lipid analysis. Bars are averages of 12 **(E&G)**, 8 to 14 **(F)**, and 6 **(H)** replicates with standard deviation, letters indicate significance at *P* < 0.05 determined by one-way ANOVA with Tukey's *post-hoc* test. LOH, LAG ONE HOMOLOG; SΔ4D, SPHINGOLIPID DELTA 4 DESATURASE; SΔ8D, SPHINGOLIPID DELTA 8 DESATURASE; GCS, GLYCOSYL CERAMIDE SYNTHASE; IPUT, INOSITOL PHOSPHORYLCERAMIDE GLUCURONOSYLTRANSFERASE; GT64, GLYCOSYL TRANSFERASE 64.

Glycosyl inositol phosphorylceramides (GIPCs) are complex phosphosphingolipids that are essential and abundant components of plant cell membranes (Tjellström et al. 2010; Cacas et al. 2016; Bahammou et al. 2024) (Fig. 1, A and B). Genetic manipulation of different plant species, including *Arabidopsis thaliana*, rice (*Oryza sativa*), and *Medicago truncatula*, has produced mutants with modified GIPC content that exhibit abnormalities in their interactions with symbiotic microorganisms (Moore et al. 2021 ), pathogen resistance (Lenarčič et al. 2017), and responses to abiotic conditions (Jiang et al. 2019). GIPC-deficient mutants also usually show obvious growth defects in the absence of environmental stresses, and knock-out mutants that lack GIPCs entirely cannot be recovered (Wang et al. 2008; Rennie et al. 2014; Ito et al. 2021). This suggests that in addition to contributing to plant interactions with their environment, GIPCs are essential for normal physiological processes and development.

Defining precise roles of GIPCs in growth and development is challenging for two reasons. First, most studies on GIPC functions have been conducted in vascular plant models. Their complex organ structures and developmental patterns can be difficult to analyze, and mutants can be non-viable when a single developmental step is blocked by a given mutation. Second, GIPCs are synthesized from ceramides, the defining lipidic backbone common to sphingolipids. Therefore, blocking steps in headgroup assembly not only results in reduced GIPC levels, but also elevated levels of free ceramides. As ceramides are potent signals that trigger programmed cell death (Berkey et al. 2012; König et al. 2022), it can be difficult to discern whether mutant phenotypes arise from substrate accumulation or from product depletion.

GIPCs have particular physical properties. They are more saturated than most membrane lipids, especially in a species such as *Physcomitrium patens* (*P. patens*), where the total acyl lipid content includes approximately 20% 18:3 (α-linolenic acid) and 20% 20:4 (arachidonic acid) (Resemann et al. 2021). In contrast, *P. patens* GIPCs typically contain saturated long-chain bases (LCBs) esterified to either saturated or monounsaturated very-long-chain fatty acid (VLCFA) moieties. Specifically, GIPCs contain predominantly C_24_ fatty acyl components compared to a roughly 50:30:20 ratio of 16:18:20 carbon fatty acids in the total lipid content. Additionally, hydroxylations on the LCB and VLCFA moieties of GIPCs can form hydrogen bonds between lipids in the membrane. These features are expected to impart increased lipid-ordered properties on their local membrane domain environment, which in turn affect the organization or function of other protein and lipid membrane components (Gronnier et al. 2016; Jaillais et al. 2024). Recent work on artificial microRNA (amiRNA) *INOSITOL PHOSPHORYLCERAMIDE SYNTHASE* (*IPCS*) transgenic lines of *A. thaliana* suggests that GIPCs drive the localized depletion of the phosphoinositide phosphatidylinositol-4-phosphate (PI4P) (Ito et al. 2021). The development and application of fluorescent probes with affinity for specific phosphoinositides have revealed myriad impacts of their distinct localizations on fundamental cell biology (Simon et al. 2014; Noack and Jaillais 2017; Markovic and Jaillais 2022). By mediating PI4P distribution, GIPCs can be expected to influence these processes as well. Further, the VLCFA moieties of GIPCs have been shown to be essential for normal endomembrane trafficking (Molino et al. 2014; Wattelet-Boyer et al. 2016), likely through a direct role in stabilizing highly-curved membranes that form during vesicle fusion (Schneiter et al. 2004; Molino et al. 2014).

Several studies have suggested that GIPCs are involved in the formation and function of plasmodesmal cell connections in plants (Zhang et al. 2022; Fougère et al. 2024). Enrichment of plasmodesma-derived plasma membrane from cell walls of *A. thaliana* suspension cell cultures revealed a higher GIPC content relative to the total plasma membrane (Grison et al. 2015). Further, mutants of *A. thaliana PHLOEM LOADING MODULATOR* (*PLM*) displayed defects in plasmodesmal development and intercellular connectivity, as well as a mild reduction in GIPC levels (Yan et al. 2019). PLM shares sequence identity and conserved motifs with *A. thaliana* IPCSs, which catalyze the committed step in GIPC synthesis. Therefore, although the biochemical activity of PLM remains uncertain, its sequence and mutant phenotype suggest that it may contribute to GIPC accumulation in a cell type- or membrane domain-specific manner.

*Physcomitrium patens* is a valuable genetic model for isolating and studying strong mutant phenotypes that severely affect growth and development. There are routine protocols for carrying out precise genome manipulation without going through a reproductive cycle (Schaefer 2001; Lopez-Obando et al. 2016; Collonnier et al. 2017); as such, it is possible to generate and cultivate mutants with severe developmental defects. Additionally, the filamentous protonemal growth and single-cell thick phyllids of moss gametophores are easy to image and monitor for phenotypes related to cell division, differentiation, and growth. Profiling *P. patens* sphingolipids with targeted lipidomics revealed the presence of GIPCs containing hexose sugars (Hex) as well as *N*-acetylated hexoses (HexNAc), within the same tissues (Resemann et al. 2021; Steinberger et al. 2021). Additionally, A-, B-, and C-series GIPCs were detected, each containing 1, 2, or 3 sugars (Hex, HexNAc, or pentose [Pent]), respectively, linked to the glucuronic acid moiety of the headgroup (Steinberger et al. 2021) (See structure, Fig. 1A, and simplified pathway, Fig. 1B).

We previously identified several GIPC-deficient *P. patens* mutants by targeting the three members of the *IPCS* gene family by CRISPR/Cas9 mutagenesis (Haslam et al. 2024). We were especially interested in (1) single, frame-shift-lesion mutants of *ipcs2*, which showed a slight decrease in Hex-GIPCs, and (2) a particular *ipcs1 ipcs2 ipcs3* triple mutant allele that showed severe reductions in Hex-GIPCs and Hex-Hex-GIPCs (*ipcst.92*). *ipcst.92* has frame-shift lesions in *IPCS1* and *IPCS3*, and a small deletion that does not disrupt the reading frame in *IPCS2*. While these single and triple mutants caused decreases in A- and B-series GIPCs with hexose headgroups, they showed only modest reductions in HexNAc-containing GIPCs. The *ipcst.92* mutant was especially unusual in that it showed no significant increase in ceramide substrate despite the GIPC product depletion, and an exponential accumulation of the direct product of IPCS activity, inositol phosphorylceramides (IPCs). To explain both findings, we previously proposed that the *ipcs2* gene product in this triple mutant is still expressed and active, but produces and accumulates the IPC product in a location where it is inaccessible for further glycosylations (Haslam et al. 2024). We also previously generated *sphinganine-C4-hydroxylase* (*s4h*) mutants by homologous recombination (Gömann et al. 2021) (also known as *sphingoid base hydroxylase*, *sbh* (Steinberger et al. 2021)). S4H hydroxylates di-hydroxy (18:0;2 or d18:0) long-chain bases (LCBs) to tri-hydroxy LCBs (18:0;3 or t18:0), which are preferentially used in the synthesis of GIPCs. Perhaps surprisingly, among the different changes in the sphingolipid profile of *s4h* mutants, we noticed a 5- to 6-fold increase in B-series GIPCs, including Hex-Hex-GIPCs and Hex-HexNAc-GIPCs, while the A-series GIPCs were not substantially altered compared to the wild type.

Together, this selection of moss mutants presents an effective toolkit to investigate the impacts of different GIPC compositions on growth and development, in a model system that is both easy to image and particularly tolerant of fundamental physiological and developmental deficiencies. We quantified macroscopic growth of gametophore colonies, cell division and differentiation in the leaf-like phyllids, and ultrastructural differences between the wild type and mutant cells. We observed strong phenotypes related to cell division in both *ipcst.92* and *s4h*, and malformation of plasmodesmata in all mutants. The physiological consequences of these defects were further investigated using intercellular motility assays with fluorescently tagged proteins and small chemical fluorescent probes. Our results give insight into the links between sphingolipid metabolism and core aspects of plant cell biology, in a bryophyte model offering a a complementary and phylodiverse comparison to *A. thaliana*.

## Results

### *ipcs* and *s4h* mutants cause substantial and distinct modifications to the GIPC profile of *P. patens*

We first confirmed the validity of metabolic deficiencies of our mutants of interest after cultivating and processing material from all genotypes in parallel under uniform growth conditions. We used mature gametophore tissues, whereas protonemal tissues were analyzed in previous characterization of *s4h* (Gömann et al. 2021). This corroborated earlier reports (Gömann et al. 2021; Haslam et al. 2024), with several substantial and statistically significant differences to GIPC profiles motivating our further interest in these mutants (Fig. 1C).

Absolute quantification of GIPCs is not possible without appropriate analytical standards. We therefore represented differences in GIPC amounts between samples as multiple reaction monitoring (MRM) peak areas normalized against a total fatty acid methyl ester (FAME) measurement. FAMEs serve as a proxy for the overall amount of acyl lipid per sample (Fig. 1C, Supplementary Fig. S1). Additionally, we expressed GIPC amounts in fold-change values relative to the wild-type measurement for that lipid class, with the wild-type value for each class of GIPC arbitrarily set to one; this provides a different perspective of the relative shift in each class (Fig. 1C). As ionization efficiencies of different GIPC classes are highly inconsistent, these approaches are meaningful for relative comparisons among genotypes, but do not reflect the quantitative profiles of the individual samples or genotypes.

We confirmed the expected sphingolipid deficiencies of the *ipcs2.1.11* mutant, as a representative *ipcs2* full loss-of-function allele. Hex-GIPCs were decreased by approximately 30% relative to wild type (Supplementary Fig. S1A), and resulted in an approximately 20% reduction of the overall GIPC levels (Fig. 1C). While the metabolic shift in GIPC synthesis observed among *ipcs2* single mutant alleles is variable and not significant (Supplementary Fig. S1), their growth phenotypes are consistent (Fig. 1D, Supplementary Fig. S2). This suggests that loss of *IPCS2*, among the three *IPCS* loci, is sufficient to disrupt the spatiotemporal control of sphingolipid homeostasis in ways that affect developmental processes yet remain subtle at the analytical level.

In the more severely-impacted *ipcst.92* triple mutant, we observed an approximately 70% reduction in Hex-GIPCs (Supplementary Fig. S1A), and similar reductions in Hex-Hex-GIPCs (Supplementary Fig. S1B) and Pent-Hex-Hex-GIPCs (Supplementary Fig. S1C), compared to wild type, while levels of HexNAc-decorated GIPCs were unchanged (Supplementary Fig. S1, D and E). Overall, this resulted in an approximate 40% reduction of total GIPCs in this mutant (Fig. 1C). The conspicuous trend of *ipcs* lesions having little or no impact on HexNAc-containing GIPCs suggests that the synthesis of these could be regulated differently than for GIPCs decorated with only hexose(s) and pentose. Notably, the metabolic phenotype of *ipcst.92* is not limited to GIPCs. As we previously reported, *ipcst.92* strongly accumulates IPCs (Supplementary Fig. S1F). Inositol phosphorylceramides (IPCs) are the direct products of IPCSs, and are metabolic intermediates that should be directly glycosylated to produce GIPCs. Whether GIPC depletion or IPC accumulation is responsible for the growth phenotype of *ipcst.92* (Fig. 1D, Supplementary Fig. S2) can be resolved by comparison to another mutant allele: The triple mutant *ipcst.46* accumulates similarly elevated levels of IPCs, but has only modest GIPC reductions compared to *ipcst.92*. Remarkably, *ipcst.46* appears morphologically indistinguishable from wild type *P. patens.* Therefore, the strong impairment of GIPC synthesis specific to *ipcst.92*, not the shared IPC accumulation that the two triple mutant alleles share, is likely responsible for the severe *ipcst.92* growth phenotype (Haslam et al. 2024). Notably, we observed no substantial difference in the free ceramide and HexCer levels of *ipcst.92* (Supplementary Fig. S1, G to J).

We also confirmed the puzzling GIPC chemotype of the *s4h* mutant, namely the dramatic increase in B-series GIPCs (6-fold increase in Hex-Hex-GIPCs, 12-fold increase in Hex-HexNAc-GIPCs, Fig. 1C, Supplementary Fig. S1, B and E), and further observed a moderate increase in C-series GIPCs (2-fold increase in Pent-Hex-Hex-GIPCs, Fig. 1C, Supplementary Fig. S1C). We also observed mild, though insignificant, reductions in A-series GIPCs in *s4h* (Fig. 1C, Supplementary Fig. S1, A and D); altogether these changes resulted in a slight increase in total GIPC levels in *s4h* relative to wild type (Fig. 1C). Directly related to the metabolic activity of S4H, in the mutant there was near-complete depletion of all sphingolipids containing an 18:0;3 LCB moiety, which were replaced with 18:0;2 LCB moieties (Supplementary Fig. S1, A to F, Fig. 1B). In the case of free ceramides, this compensation was incomplete, with free ceramides accumulating to only approximately 20% of the wild-type level (Supplementary Fig. S1, G and H). Additionally, HexCer levels in *s4h* nearly doubled compared to wild type (Supplementary Fig. S1I), and strikingly, contained a more diverse fatty acid profile (Supplementary Fig. S1J). It is not obvious which of these metabolic phenotypes is causal for the severe growth phenotype of *s4h*. Similar metabolic and developmental phenotypes were reported for CRISPR/Cas9-generated *s4h*/*sbh* mutant alleles (Steinberger et al. 2021).

In summary, the sphingolipid chemotype of the *ipcs2* single mutant is restricted to a mild depletion of GIPCs. The *ipcst.92* mutant has a more complex metabolic phenotype, however its developmental phenotype seems to depend on the depletion of GIPCs. In contrast, growth and development of *s4h* may be impacted by severe changes in its LCB, ceramide, HexCer, and GIPC profiles.

### The *ipcs2.1.11*, *ipcst.92*, and *s4h* mutants have characteristic morphological phenotypes

All three mutants have obvious morphological phenotypes (Fig. 1D, and Supplementary Fig. S2 for images of additional mutant alleles). These were quantified based on images of 2-month-old gametophores of different genotypes grown together on the same plates. Gametophore colony area is significantly reduced in all three genotypes, with a near 50% reduction in *ipcs2.1.11*, and approximately 90% reductions in both *ipcst.92* and *s4h* (Fig. 1E). *ipcs2.1.11* overall appears morphologically similar to wild type, but smaller. In contrast, *ipcst.92* and in particular *s4h,* are both dwarf and morphologically unusual. Reduction of gametophore colony expansion in *ipcst.92* and *s4h* (Fig. 1E) was more severe than reduction of individual phyllid size (Fig. 1F). This discrepancy must be due to other factors that affect colony size being modified in the mutants, such as gametophore formation rate or cauloid elongation, and gives the two severe mutants a dense, round appearance compared to the stellate wild type and *ipcs2.1.11*. The altered form of *ipcst.92* and *s4h* can be quantified as circularity, which was increased nearly 10-fold in these two mutants compared to wild type (Fig. 1G). Additionally, the % dry mass of *ipcst.92* and *s4h* was significantly increased relative to wild type, by approximately 30% (Fig. 1H), presumably due at least in part to the relative increase in apoplastic vs. symplastic material that would occur with reduced cell size, and corresponding increased surface area:volume ratio.

### Cell division, expansion, and differentiation are all altered compared to wild type in mutant phyllids

**Figure 2.**
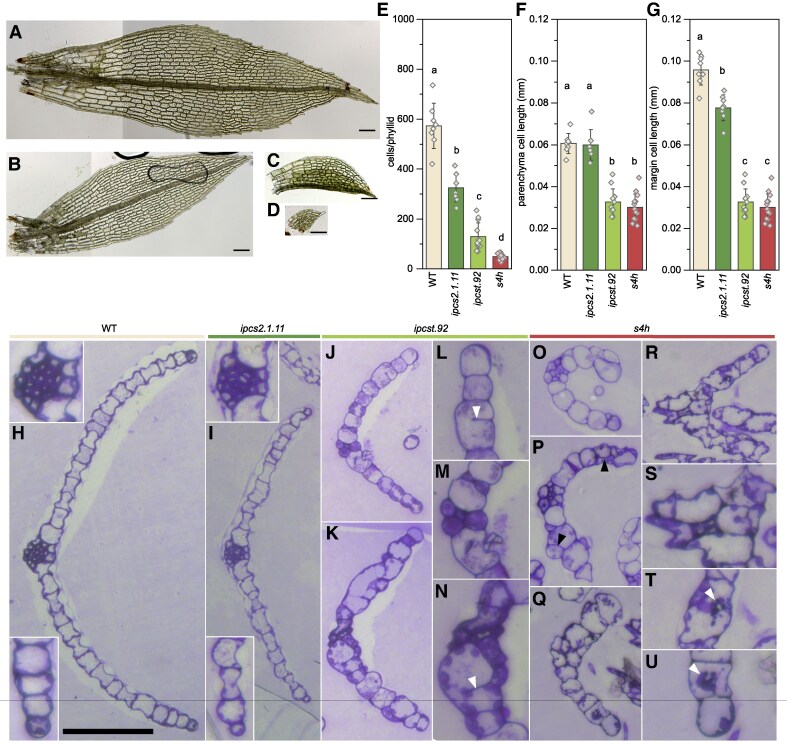
Representative images of whole and sectioned phyllids. Images of whole wild type **(A)**, *ipcs2.1.11*  **(B)**, *ipcst.92*  **(C)**, and *s4h*  **(D)** were used for phyllid and cell measurements **(E-G)**. All scale bars represent 0.1 mm. Images are stitched from multiple snapshots taken of different phyllid regions, and in different focal planes. **E)** Number of cells per phyllid. **F)** Length of phyllid parenchyma cells. **G)** Length of phyllid margin cells. Measurements in F and G are the same for *ipcst.92* and *s4h*, as cell types could not be clearly distinguished. Bars are averages of 8 to 14 phyllids with standard deviation, letters indicate significance at *P* < 0.05 determined by one-way ANOVA with Tukey's *post-hoc* test. Wild type **(H)**, *ipcs2.1.11* single mutant **(I)**, *ipcst.92* triple mutant **(J-N)**, *s4h* mutant young **(O, P)** and mature phyllids **(Q-U)**. Note there is a small region of overlap between O and P. The scale bar in H corresponds to 100 *µ*m in the overviews **(H, I, J, K, O-R)**; and to 50 *µ*m in the insets of **(H, I)**, and in **(L-N, S-U)**, which show enlarged details of the hadroms and the phyllid margin cells, as well as mutant peculiarities. L and M are magnified from J, N is magnified from K, S and T are magnified from R, and U is magnified from Q. Incomplete cell walls are marked with white arrowheads in **(L, N)**, oblique and periclinal divisions with black arrowheads in **(P)**, and irregularly shaped, densely stained material is marked with white arrowheads in **(T**, **U)**.

Phyllid histological features and differentiation into distinct cell types (Dennis et al. 2019; Lin et al. 2021) were next observed in surface view (Fig. 2, A to G), and in crystal violet-stained semi-thin cross sections (Fig. 2, H to U). There are several distinct cell types in phyllids. Phyllid margin (or edge) cells have an elongated form and protrude slightly at the apical end, producing a rounded serration along the margin. Stereid and hydroid cells in the hadrom (a “midrib” in analogy to vascular plants), are elongated, straight, and stereids have thicker cell walls than the other cell types. The remaining cells between the hadrom and margin are considered here simply as parenchyma, and vary substantially in size, but generally have a shorter aspect ratio than the margin, stereid, and hydroid cells, and are more densely filled with chloroplasts. In some literature, parenchyma cells are further sub-divided into base and tip cells, and are distinguished based on their shape and size (Dennis et al. 2019; Lin et al. 2021).

Dramatic reductions were clear in phyllid cell numbers for all mutants, with *ipcs2.1.11* reduced to 55%, *ipcst.92* to 20%, and *s4h* to 10% of the cell number per phyllid compared to the wild type (Fig. 2E). Average cell length was calculated by measuring individual phyllids and dividing the length by the number of cells in a column spanning the length of the phyllid (Fig. 2, F and G). We could clearly distinguish between parenchyma and margin cells in the wild type and in *ipcs2.1.11*, and so these cell types could be measured separately. In *ipcst.92* and *s4h*, there was no consistent, distinct shape to cells at the phyllid margin; additionally, cell columns within the phyllid were difficult to track. Therefore, only an edge row of cells was used for quantification of these two genotypes. By comparison to wild-type parenchyma or margin cells, *ipcst.92* and *s4h* phyllid cells were reduced to between 30 and 50% of the normal length (Fig. 2, F and G). For *ipcs2.1.11*, only margin cells were approximately 15% shorter (Fig. 2G), whereas parenchyma showed no significant difference (Fig. 2F). Overall, the reduction in phyllid size was primarily due to reduced cell number for *ipcs2.1.11*, whereas for *ipcst.92* and *s4h*, both cell length and cell number were substantially reduced.

In median sections through wild-type phyllids (Fig. 2H), the water-conducting hydroids and the thick-walled stereids located in the hadrom (upper inset in Fig. 2H) separated the two halves of the phyllid, which consisted of parenchyma and margin cells at the outer edge (lower inset in Fig. 2H), in a single cell layer. Anticlinal cell walls were regularly-spaced and occurred at near-perfect right angles to the phyllid surface. The phyllid anatomy and histology of the *ipcs2.1.11* single mutant (Fig. 2I) did not differ considerably from the wild type, with an easily discernible hadrom (upper inset in Fig. 2I), differentiated margin cells, and normally-shaped but fewer parenchyma cells (lower inset in Fig. 2I).

Phyllid anatomy was severely affected in the *ipcst.92* triple mutant, with obvious developmental defects in the hadroms, which were composed of fewer, often wider cells (Fig. 2, J, K and M). Only few, if any, cells developed the characteristically thick stereid cell walls. Apart from the reduced parenchyma cell numbers (Fig. 2, E to G, J and K), further observation of *ipcst.92* phyllid sections hinted at a general defect in the control of cell division (Fig. 2, L and N): we often observed strikingly wide cells with incomplete anticlinal walls, which extended from one side of the parental cell wall and ended bluntly in the cell (white arrowheads in Fig. 2, L and N), thereby failing to separate adjacent cells completely from each other.

Phyllids of the *s4h* mutant also showed severe histological defects, with either ambiguous or indiscernible hadroms, and wide variation in parenchyma cell width and shape throughout the phyllid cross sections (Fig. 2, O to S). They had striking oblique and periclinal division planes (black arrowheads in Fig. 2P), leading to partially-multilayered phyllids. Another characteristic feature of the *s4h* mutant phyllid cells was irregularly shaped, tufted aggregates of material, darkly stained with crystal violet, whose direct attachment to the cell walls could be seen within serial sections (white arrowheads in Fig. 2, T and U).

Reduced size of the mutant phyllids and reduced cell number per phyllid (Fig. 2, A to E) could be easily correlated to reduced numbers of cell columns per phyllid in these cross sections (Fig. 2, H to U), approximately 15 per phyllid half in wild type, 10 in *ipcs2.1.11*, 8 in *ipcst.92*, and 6 in *s4h*. Perhaps surprisingly, the reductions in average cell length measured in whole phyllids (Fig. 2, F and G) did not correlate with widths observed in semi-thin sections (Fig. 2, H to U). We infer that whatever factors caused stunted apical-proximal cell elongation in *ipcst.92* and *s4h* did not substantially impact lateral cell expansion.

### TEM investigations reveal additional unique phenotypes in *ipcs* and *s4h* mutants

**Figure 3.**
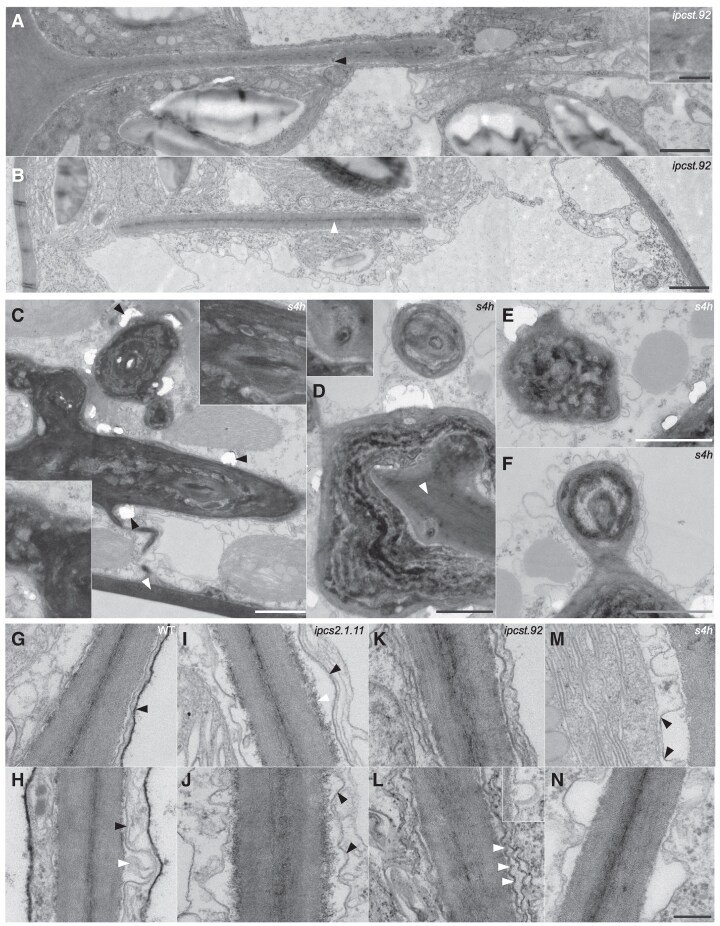
Ultrastructural peculiarities observed in the mutants via transmission electron microscopy. **A, B)** TE micrographs showing incomplete cell walls, which end bluntly in mature phyllid cells of *ipcst.92*, and exhibit a regular layering of the middle lamellae (white arrowhead in **B)** and the subsequent cell wall layers as well as plasmodesmata (black arrowhead in **A**, and magnified inset). Analysis of serial light microscopy images revealed when apparently non-attached incomplete walls were connected to the outer cell wall in other planes of section. The image shown in panel B is a composite figure. **C-F)** TE micrographs of cytosolic regions of *s4h* mutant cells with irregularly-shaped, tufted aggregates. Attachment to the outer cell walls can be seen in some sections (white arrowheads in **C** and **D)**, which included plasmodesmata **(D**, inset**)**. The depositions usually had a layered substructure composed of both light and dark materials **(C**, insets). They likely had a hard consistency, since ultrathin sections often cracked in the vicinity of the aggregates (black arrowheads in **C)**. All scale bars in **(A-F)** represent 1 *μ*m, inset have a two-fold magnification, except inset in A where the scale bar represents 200 nm. Note there is a small region of overlap between E and F. **G**-**N)** Representative TE micrographs showing peculiarities of the attachment of the plasma membrane to the cell wall. In wild type **G, H)** the plasma membrane lines the cell wall closely (black arrowhead in **H)**, and single vesicles were regularly found to fuse with the plasma membrane (white arrowhead in **H)**. The tonoplast membrane appeared unusual in that it was more electron-dense (black arrowhead in **G)** than is typically observed in wild type *P. patens* (Reute), and in the mutants from this sample set. In *ipcs2.1.11*  **I, J)**, the plasma membrane had a wavy shape and was detached from the cell wall over wide areas (black arrowheads in **I, J)**. The cell wall appears unusual in that its youngest layers were irregularly fringed (white arrowhead in **I)**. The plasma membrane of *ipcst.92*  **(K, L)** was also wavy and detached from the cell wall over large areas, with more frequent but smaller membrane bends (white arrowheads in **L)**. The inset in **(L)** shows the fusion of a small vesicle with the plasma membrane. The shape of the plasma membrane of the *s4h* mutant **(M, N)** resembles that of the *ipcs2.1.11* single mutant, but detached membrane areas were more common, more deeply invaginated, and often included particularly straight and angular membrane sections with higher electron density (black arrowheads in **M)**. Scale bar for **(G-N)**, shown in **(N)**, is 200 nm.

We sought to better understand the unusual phenotypes observed via light microscopy by TEM. Observation of the blunt-ended anticlinal cell walls in the *ipcst.92* mutant (Fig. 3, A and B) revealed the presence of plasmodesmata (black arrowhead in Fig. 3A and inset), as well as regular internal layering and the presence of a middle lamella (white arrowhead in Fig. 3B). Structurally, they were not obviously different from other mature cell walls in the *ipcst.92* mutant or wild-type phyllids. The surrounding cytoplasm was inconspicuous and lacked vesicles and phragmoplast microtubules that would have been present in the case of ongoing cytokinesis. Altogether, this suggests that *ipcst.92* fails to organize cell plate attachment to the parental cell wall to complete cytokinesis.

The aggregations deposited onto cell walls in *s4h* were also further investigated by TEM: They appeared irregular in form, mostly electron-dense, and had an intricate, layered sub-structure including some electron-lucent regions (Fig. 3, C to F). The ultrathin sections were often cracked directly adjacent to these aggregates (black arrowheads in Fig. 3C), suggesting that they may have an especially hard consistency. They were often observed in direct contact with cell walls (white arrowheads in Fig. 3, C and D). The overall form of these depositions was reminiscent of aniline blue-stained structures previously observed surrounding cross walls in protonemal filaments of *s4h*, inferred to be abnormal callose depositions (Gömann et al. 2021; Ye and Zhong 2022), as well as red-colored intracellular aggregates previously observed in phyllids of *s4h* (*sbh*) (Steinberger et al. 2021). The presence of both of these accumulations was confirmed in gametophores grown under our experimental conditions via light and fluorescent microscopy (Supplementary Fig. S3, A to C). Aniline blue-stained and red-colored structures generally did not co-localize (Supplementary Fig. S3C). Yet, we did not observe two different types of aggregates in the TEM sections, but rather found structures composed of at least two different materials.

Due to the chemical impact of GIPCs on plasma membrane-cell wall adherence (Voxeur and Fry 2014), we attempted to observe contact between these structures in TE micrographs of our GIPC-deficient mutants (Fig. 3, G to N, Supplementary Fig. S4). In wild type, (Fig. 3, G and H, Supplementary Fig. S4, A and B), there were regions with close, parallel alignment between the plasma membrane and cell wall (black arrowhead in 3H), as well as vesicle contact sites where the plasma membrane is invaginated (white arrowhead in 3H). The tonoplast membrane seemed to be associated with granular vacuolar material and appeared unusually thick and darkly-stained (black arrowhead in 3G), even by comparison to similarly-treated *P. patens* (Reute) phyllids (Wegner and Ehlers 2024). In all three GIPC mutants, plasma membrane detachment from the cell wall could easily be observed, but with distinct characteristics in each mutant (Fig. 3, I to N, Supplementary Supplementary Fig. S4, C to L). In *ipcs2.1.11* (Fig. 3, I and J, Supplementary Fig. S4, C and D), detachment occurred over large regions, and the plasma membrane had a wavy appearance (black arrowheads in Fig. 3, I and J). The inner layers of the cell wall often had an electron-dense fringe (white arrowhead in 3I). Tonoplast membranes with similar electron density to the wild type were only occasionally observed in *ipcs2.1.11*, otherwise this was not observed in the mutants (Supplementary Fig. S4D). In *ipcst.92* (Fig. 3, K and L, Supplementary Fig. S4, E to G) large expanses of wavy, detached plasma membrane were obvious (white arrowheads in 3L); here the waves had a markedly shorter length, producing a densely rippled appearance, and the invaginations were not as deep as in the other two mutants. In *s4h* (Fig. 3, M and N, Supplementary Fig. S4, H to L), plasma membrane detachments were also frequent (black arrowheads in Fig. 3M). Remarkably, the membrane curvature often was sharp to the point that it appeared angular (Fig. 3M), and the invaginations were deepest here among the three mutant genotypes. These angular membrane sections appeared to have higher electron density within the otherwise weakly contrasted plasma membrane. Beyond these plasma membrane-cell wall adherence phenotypes, organelles and cellular compartments in phyllid parenchyma cells of the mutants largely resembled those of the wild type, with a few differences (Supplementary Fig. S4, also see Discussion).

### Plasmodesmal ultrastructure of phyllids is impacted by modification of GIPC content

**Figure 4.**
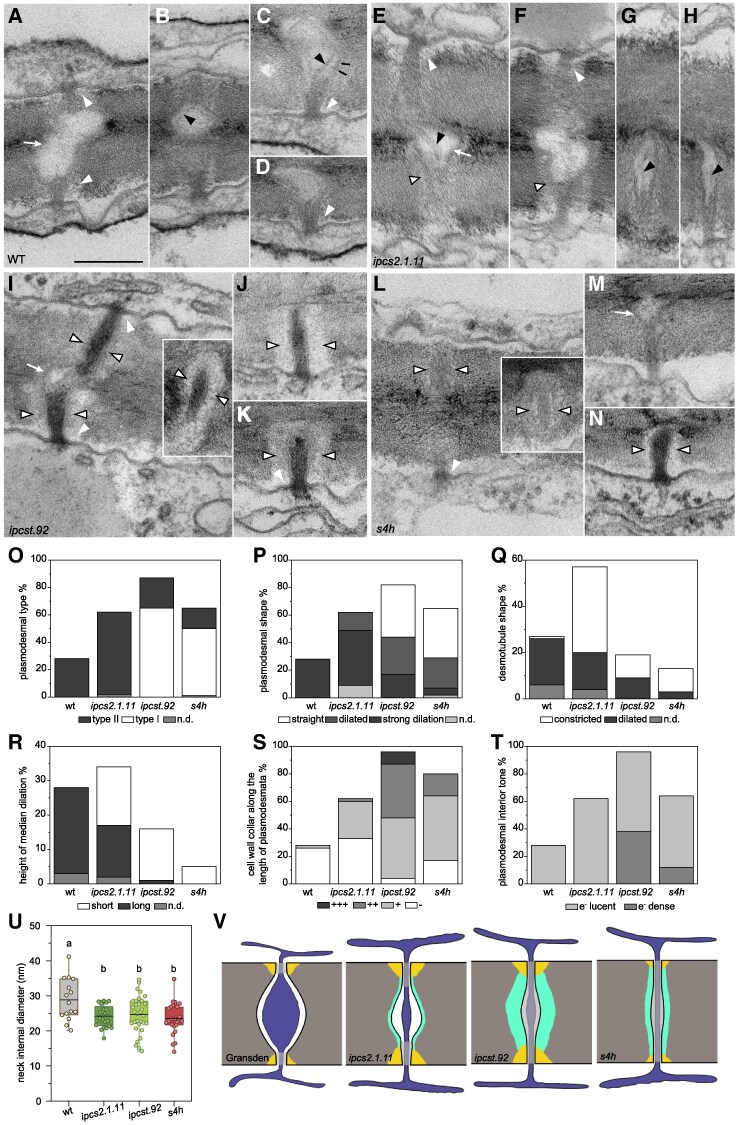
GIPC deficiencies are associated with changes to plasmodesmal ultrastructure. Transmission electron micrographs **(A-N)**, quantification **(O-U)**, and schematic drawings **(V)**, showing the typical structures of plasmodesmata observed in anticlinal cell walls of mature phyllids. Micrographs show the wild type **(A-D)**, the *ipcs2.1.11* single mutant **(E-H)**, the *ipcst.92* triple mutant **(I-K)**, and the *s4h* mutant **(L-N)**. The scale bar shown in A represents 200 nm in all TEM images. White arrowheads point at callose deposits constricting the neck regions; white arrows mark strong median dilations; black arrowheads point at the membranes of dilated **(B, C)** or constricted **(E, G, H)** desmotubules; open arrowheads indicate electron-lucent cell wall collars; and black lines in C point at rarely visible spoke-like proteins spanning the cytosolic sleeve. Graphs in **(O-T)** depict the percentages of plasmodesmata showing distinct traits of morphological characters. Only those plasmodesmata in which the indicated character was visible in the plane of section were taken into account, with Q (desmotubule shape) referring only to the type II (-like) plasmodesmata in the respective line, and **(R)** (median dilation height) considering only those plasmodesmata showing strong median dilations. **U)** Neck internal diameter in nm, measured at the narrowest point observed. Thick black lines represent the mean, boxes represent 25 to 75% distribution of the data, whiskers the minimum and maximum values, letters indicate significance at *P < 0.05 determined by one-way ANOVA with Tukey's *post-hoc* test. In the schematic drawings **(V)** all membranes are shown in black, the cell wall is depicted in grey, the ER is illustrated in dark blue, white shows the cytosolic sleeve between the plasma membrane and the central desmotubule. A grey overlay marks regions in which plasmodesmal substructures could not clearly be seen (i.e. necks and type I plasmodesmata). Yellow colour indicates callose deposits at the constricted plasmodesmal orifices, and aquamarine depicts electron-lucent cell wall collars surrounding the plasmodesmata.

TE micrographs were also examined for alterations of plasmodesmal structures of the mutants. Although median phyllid sections were compared between genotypes, the strong developmental phenotypes of the *ipcst.92* and *s4h* mutants introduced some complexity to comparisons given that plasmodesmal structure and function is known to progress alongside cell maturation. The specifics of this process were recently described for *P. patens* Reute (Wegner and Ehlers 2024). Similar to angiosperms (Nicolas et al. 2017), young, narrow type I plasmodesmata typically have a uniform diameter of approximately 25 nm over their length, and internal structures cannot be distinguished. These plasmodesmata have a relatively high size exclusion limit (SEL) of at least 27 kDa, which allows the movement of macromolecules between cells. In contrast, in older type II(-like) plasmodesmata characteristic of mature cells, internal substructures can clearly be seen, including the central ER-derived desmotubule and the cytosolic sleeve between the plasma membrane and desmotubule. These type II(-like) plasmodesmata are wider at approximately 45 nm in the dilated region located in the median cell wall layers (white arrows in Fig. 4, A to N), and show constricted “necks” only at the orifices (white arrowheads in Fig. 4, A to N), where the plasma membrane and desmotubule membrane (black arrowheads in Fig. 4, A to N) are still locally difficult to distinguish. Type II(-like) plasmodesmata have a much lower size exclusion limit, approximately 1 kDa in *A. thaliana*, and therefore restrict the intercellular transport between older cells to small molecules (Nicolas et al. 2017; Wegner and Ehlers 2024).

All plasmodesmata observed in mature phyllids of the wild type (Fig. 4, A to D, n = 28) were classified as type II(-like) (Fig. 4O), since the desmotubule and the cytosolic sleeve could be clearly discerned in the median plasmodesmal regions, which always had a strongly-dilated shape (Fig. 4P). In contrast to many characterized angiosperms, the desmotubule was also usually dilated in this region (Fig. 4Q), and spoke-like structures linking the desmotubule membrane to the plasma membrane could sometimes be observed (Fig. 4C, indicated by black lines). The median dilations of the wild-type plasmodesmata were always long (Fig. 4R) and covered more than half of the cell wall thickness. Cell wall collars surrounding the median region of the plasmodesmata were occasionally observed, in 7% of the wild-type plasmodesmata (Fig. 4S, open arrowheads indicate electron-lucent cell wall collars in 4E-N). The interior of the cytosolic sleeves and desmotubules were electron-lucent (Fig. 4T), and short, constricted, electron-dense neck regions at the plasmodesmal orifices were surrounded by moderate callose deposits (Supplementary Fig. S3D), restricting the average minimal inner diameters of the plasmodesmal neck regions to 28.9 ± 6.0 nm (Fig. 4U). Thus, plasmodesmal characters of wild type (Gransden) phyllids resemble those described previously (for mature wild-type Reute phyllids) (Wegner and Ehlers 2024). However, at 0.158 plasmodesmata/µm^2^ cell wall (Table 1), the observed plasmodesmal density was substantially lower than in the Reute line (0.744 plasmodesmata/µm^2^ cell wall (Wegner and Ehlers 2024)).

**Table 1.** Plasmodesmal densities (ρ(PD)) of the different GIPC-mutants based on plasmodesmal counts via TEM

Genotype	nWalls	nPD	PD/wall	Total wall length	PD/µm	ρ(PD)
WT	96	28	0.292	1,269.494	0.022	0.158
*ipcs2.1.11*	82	62	0.756	799.772	0.078	0.620
*ipcst.92*	90	93	1.033	915.258	0.102	0.813
*s4h*	166	64	0.386	1,699.678	0.038	0.301

The densities (PD/µm^2^ cell wall) were calculated as described in (Wegner and Ehlers 2024) based on the total number of plasmodesmata and the total cell wall length (µm). These values were gathered from at least 82 walls per genotype, which were observed in serial sections of one or two (*s4h*) biological replicates per genotype.

In the *ipcs2.1.11* single mutant, plasmodesmal structure was moderately affected (Fig. 4, E to H, *n* = 62). Observed plasmodesmata were still consistently type II(-like) (Fig. 4O), with visible internal substructures and dilated median regions (Fig. 4P). However, in 21% of the plasmodesmata, the median region was not as strongly dilated as in the wild type. *ipcs2.1.11* plasmodesmata also differed in that their desmotubules were mostly constricted (in 65% of type II(-like) plasmodesmata) (Fig. 4Q). Additionally, *ipcs2.1.11* plasmodesmata were often surrounded by narrow, electron-lucent cell wall collars (in 47% of plasmodesmata) (Fig. 4S; open arrowheads in Fig. 4, E to N). While callose deposits at the neck regions were only slightly stronger than with the wild type (Supplementary Fig. S3D) the average minimal inner diameter of the neck regions was significantly reduced compared to the wild type (24.2 ± 3.1 nm; Fig. 4U), and the dilated median region did not cover half of the cell wall thickness, with 50% of those plasmodesmata showing strong dilations (Fig. 4R). The determined plasmodesmal density of 0.620 plasmodesmata/µm^2^ cell wall (Table 1) was about four times higher than in the wild type (Gransden) from this study, and comparable to the published data for wild-type Reute.

An even higher density of 0.813 plasmodesmata/µm^2^ cell wall (Table 1) was found in the *ipcst.92* triple mutant (Fig. 4, I to K, *n* = 87), whose plasmodesmata differed clearly from the wild type, since 75% (Fig. 4O) failed to undergo the transition from type I into type II(-like) and did not show internal substructures. Among the few type II(-like) plasmodesmata, 53% had a constricted desmotubule (Fig. 4Q). 46% of the *ipcst.92* triple mutant plasmodesmata had a straight morphology with an almost constant diameter over their entire length, and did not develop any dilations in the median regions of the cell wall (Fig. 4P). Only 21% of the *ipcst.92* plasmodesmata had strong median dilations resembling those of the wild type (Fig. 4P), but in most (94%) of these, the median dilations did not cover half of the cell wall thickness (Fig. 4R). Neck regions exhibited only slightly enhanced callose deposits (Supplementary Fig. S3D), but the average minimal inner diameter of the neck regions was significantly reduced compared to the wild type (24.7 ± 5.3 nm; Fig. 4U). One of the most striking features of *ipcst.92* triple mutant plasmodesmata was an electron-dense interior observed with 40% of plasmodesmata (Fig. 4, I to K and T). This dense interior is often seen in young type I plasmodesmata (Nicolas et al. 2017). Additionally, electron-lucent cell wall collars were observed encasing 96% of the analyzed plasmodesmata in *ipcst.92* (Fig. 4, I to K and S), a striking feature that was rare and far more subtle in the wild type (Fig. 4S).

Plasmodesmata of the *s4h* mutant (Fig. 4, L to N, *n* = 65) largely resembled those of *ipcst.92*, with 75% keeping type I morphology in the mature state (Fig. 4O). A constricted desmotubule was found in 77% of the rare type II(-like) plasmodesmata (Fig. 4Q). The majority of plasmodesmata in the *s4h* mutant (55%) had a straight morphology without any median dilation, and only 8% of mutant plasmodesmata had strong median dilations (Fig. 4P), none of which covered half of the wall thickness (Fig. 4R). Neck regions had slightly smaller callose deposits than the wild type (Supplementary Fig. S3D); nevertheless, the average minimal inner diameter of the neck regions was significantly reduced compared to the wild type (23.5 ± 4.5 nm; Fig. 4U). An electron-dense interior was occasionally observed with 19% of plasmodesmata (Fig. 4T), and moderate electron-lucent cell wall collars were found around 79% of the analyzed plasmodesmata (Fig. 4S). An additional peculiarity of the *s4h* mutant was the low electron density of the plasma membrane; this is seen in plasmodesmata (Fig. 4, L and M), but also generally throughout the plasma membrane (Fig. 3, M and N, Supplementary Fig. S4, H, I and K). Further, in contrast to both *ipcs* mutants, the plasmodesmal density of the *s4h* mutant was only 0.301 plasmodesmata/µm^2^ cell wall (Table 1) and thus reaches only twice the value observed with the wild type.

In summary (Fig. 4V), mutants with severe shifts in GIPC content failed to form normal type II(-like) plasmodesmata, and instead exhibit type I(-like) plasmodesmata with electron-dense interiors. Formation of dilated median plasmodesmal regions was impaired in all mutants, with desmotubule dilation being especially impaired relative to the wild type. The severity of the dilation phenotype increased with the overall strength of the mutant phenotype. Instead of median dilations, pronounced cell wall collars were visible around the mutant plasmodesmata. Compared to wild-type plasmodesmata, inner neck diameters were likewise significantly reduced in all mutants under investigation.

### Incomplete cell division in *ipcst.92* allows free cytosolic and ER luminal movement between phyllid cells, and plasmodesmal trafficking of macromolecules is restricted in *s4h*

**Figure 5.**
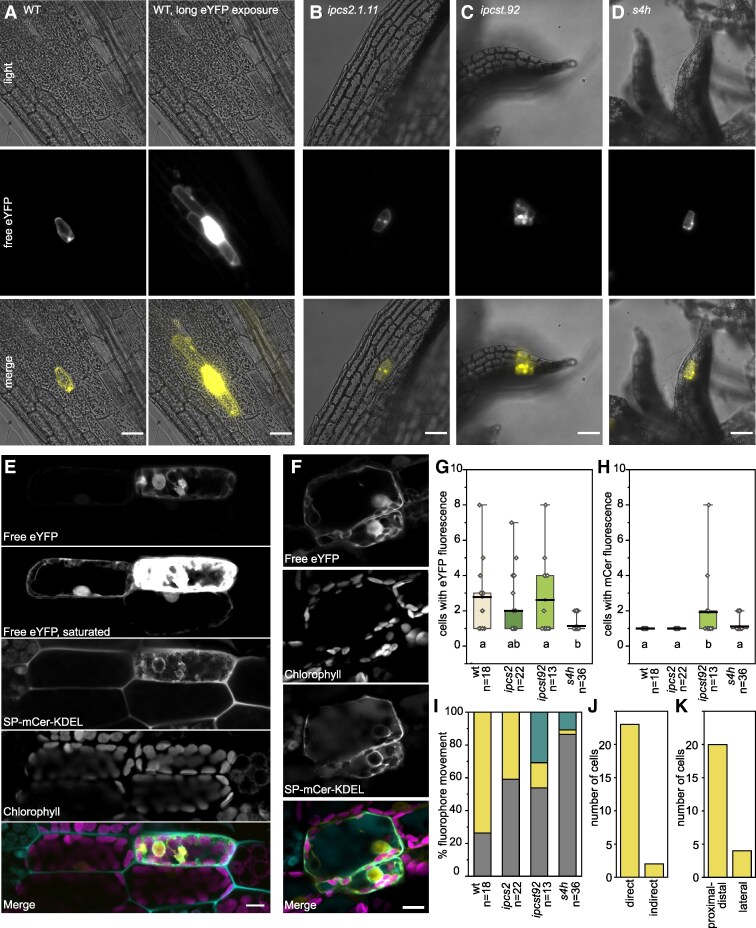
Intercellular connectivity is altered in *ipcst.92* and *s4h* mutant phyllids. **A-D)** Representative images of free enhanced yellow fluorescent protein (eYFP) distribution after bombardment in wild type **(A)** (right side is the same bombardment as left. imaged with a longer exposure time), *ipcs2.1.11*  **(B)**, *ipcst.92*  **(C)**, and *s4h*  **(D)**. Gametophore cultures were four weeks old for bombardment, and imaged 2 to 3 days after bombardment. **E)** Representative image of weak eYFP diffusion in wild type, presumably through plasmodesmata, and no intercellular diffusion of ER-luminal aspartic protease signal peptide-mCerulean-KDEL (SP-mCer-KDEL). **F)** Representative image of strong eYFP and SP-mCer-KDEL diffusion in *ipcst.92*, presumably due to movement through incomplete cell walls. **G)** Number of directly adjacent cells in clusters with eYFP signal, regardless of intensity. **H)** Number of directly adjacent cells in clusters with SP-mCer-KDEL signal. For both fluorophores, the bombarded cell is counted as *n* = 1, and only bombardments in which both fluorophores were expressed in the bombarded cell were included in the quantification. Thick black lines represent the mean, boxes represent 25 to 75% distribution of the data, whiskers the minimum and maximum values. Letters indicate significance at *P* < 0.05 determined by one-way ANOVA with Tukey's *post-hoc* test. **I)** Frequency of eYFP and SP-mCer-KDEL patterns after individual bombardment events; grey indicates fluorophores only detected in a single cell, yellow indicates the eYFP marker is detected in multiple adjacent cells while SP-mCer-KDEL is present in only a single cell, and turquoise indicates both the eYFP marker and the SP-mCer-KDEL are detected in multiple adjacent cells. **J)** Frequency of movement of eYFP in the wild type directly from the individual bombarded cell to its direct neighbours vs. movement to cells not in direct contact with the bombarded cell. **K)** Frequency of movement of eYFP in the wild-type in different directions relative to the individual bombarded cell. eYFP movement in larger cell clusters, where the source of the fluorophore was ambiguous, were excluded from the analysis. Scale bars are 50 *μ*m in A-D, 10 *μ*m in E, F.

To investigate the consequences of incomplete cell division and altered plasmodesmal structure in the mutants, we bombarded mature gametophores of 4-week-old cultures with fluorescent markers and observed and quantified their intercellular movement. Expression constructs with free enhanced yellow fluorescent protein (eYFP) (27 kDa) for cytosolic tracking and mCerulean (mCer), with an N-terminal signal peptide from aspartic protease and C-terminal ER retention signal (KDEL) (Mueller and Reski 2015) (SP-mCer-KDEL) (27 kDa) for luminal ER labeling were bombarded into all four genotypes that were co-cultivated on the same plates for four weeks. Expanded phyllids, approximately # 6 to 12 from the gametophore apex, were used for imaging two to three days post-transformation (Fig. 5). Bombardments were imaged with fluorescence microscopy for observation within a broader cellular environment (Fig. 5, A to D), and confocal laser scanning microscopy for visualization of subcellular features in individual bombarded cells (Fig. 5, E and F).

To ensure consistency of phenotypes in CRISPR/Cas9 mutants containing different mutant lesions, *ipcs2.1.3* and *ipcs2.1.16*, which are similar to *ipcs2.1.11* in having frameshift lesions early in the *IPCS2* coding sequence, were included. We also included *ipcst.92 replica#31*, which has different lesions in *IPCS1* and *IPCS3* from those in *ipcst.92*, but all of which cause early frameshift lesions in these genes (Supplementary Fig. S2). The lesions in *IPCS2* in both alleles are identical, as this lesion was introduced into a double *ipcs1 ipcs3* background by homologous recombination of the gene cloned from the original *ipcst.92* in order to generate *ipcst.92 replica #31.* As no obvious differences were observed among *ipcs2* alleles, and the unique phenotypes of *ipcst.92* were observed with similar frequency in both alleles examined, results from all alleles were included for phenotype quantification (Fig. 5, G to I). The experiment was repeated twice on older (five to eight week old) gametophores and similar trends were observed (Supplementary Fig. S5, A to C).

In the wild type (Fig. 5, A and E), eYFP fluorescence was restricted to a single cell after 26% (5/19) of bombardment events; the remaining 74% (14/19) of bombardments resulted in some eYFP fluorescence in 1 to 7 additional adjacent cells (Fig. 5, G and I). Fluorescence in adjacent cells was consistently very weak, with eYFP only detectable upon enhancement of the signal to the point of saturation in the bombarded cell (Fig. 5E). In contrast, SP-mCer-KDEL was always exclusive to the bombarded cell (Fig. 5, E and H). This pattern of fluorophore movement suggests that it is mediated by plasmodesmata. Restricted luminal ER signal fits with previous observations that the desmotubule lumen does not allow for the transport of macromolecules larger than 3 to 10 kDa (Crawford and Zambryski 2000; Li et al. 2021). The spread of eYFP to adjacent cells can be attributed to a relatively young age of these cells and associated high SEL of younger plasmodesmata (Wegner and Ehlers 2024). We infer that in wild type, eYFP is able to pass through plasmodesmata of adjacent cells, with a limited capacity that is dependent on the SEL of plasmodesmata and developmental stage of the bombarded gametophore colony, phyllid, and individual cell. When intercellular movement of eYFP was observed, it was limited most of the time to cells in direct contact with the bombarded individual cell (23/25 neighbouring cells) (Fig. 5J). Moreover, there was a strong tendency for movement in the apical-proximal axis of the phyllid (20/24 neighbouring cells) compared to lateral movement across the phyllid (Fig. 5K).

Compared with the wild type, similar patterns of fluorophore spread were observed in *ipcs2* single mutants (Fig. 5B), with weak cytosolic free eYFP movement observed following approximately 50% of bombardment events (Fig. 5, G and I), and no intercellular ER-luminal SP-mCer-KDEL motility (Fig. 5, H and I) (ratios were 2:3, 6:5, and 5:1 for no movement to eYFP movement for *ipcs2.1.3, ipcs2.1.11,* and *ipcs2.1.16,* respectively).

In *ipcst.92*, cytosolic eYFP fluorescence was also restricted to a single cell following approximately 50% of bombardment events, and shared in the remaining 50% (Fig. 5, C and I). However, the appearance of eYFP-fluorescent cell clusters in *ipcst.92* was unmistakably distinct from the wild type, in that the clusters were uniformly bright with eYFP to the extent that the bombarded cell could not be identified within a cluster (Fig. 5F). This suggests that in the *ipcst.92* mutant there is unregulated cytosolic eYFP movement within fluorescent cell clusters. Further, unlike other genotypes tested, in *ipcst.92* the cells adjacent to the bombarded cell regularly exhibit fluorescence of not only cytosolic eYFP, but also the SP-mCer-KDEL marker (Fig. 5, C and F). The intercellular spread of the SP-mCer-KDEL, which is well above the size of desmotubule-mobile molecules, together with the even intensity of the cytosolic eYFP signal, suggest that there is free, non-plasmodesmal connectivity of both cytosol and ER lumen in *ipcst.92*, most likely via the incomplete anticlinal cell division walls observed in Figs. 2, L and N, 3, A and B. Additionally, eYFP was observed to move beyond the cells expressing SP-mCer-KDEL, indicating that plasmodesmal macromolecular transport was also retained in *ipcst.92.* (Ratios were 2:0:2 and 5:2:2 for no movement, eYFP movement, and movement of both fluorophores for the original *ipcst.92* and *ipcst.92*  *replica #31*). Though analysis was primarily focussed on gametophores for the present study, replication of the biolistic bombardments on protonema did not produce any evidence of similarly-incomplete cell division in this tissue (Supplementary Fig. S5, D and E).

In the tiny *s4h* phyllids, eYFP fluorescence was generally restricted to a single bombarded cell (Fig. 5D). However, it was difficult to identify healthy bombarded cells with healthy neighbouring cells, which could be analyzed without interference from autofluorescence of damaged cells. Following several replicates of this experiment (Supplementary Fig. S5, A to C) we focused screening especially on *s4h* (wt *n* = 18, *ipcs2 n* = 22, *ipcst.92 n* = 13, *s4h n* = 36) and finally observed movement of both fluorophores in four separate pairs of cells, as well as weak diffusion of only cytosolic eYFP to one adjacent cell in one additional bombardment event (Fig. 5, G to I). This trend of consistent reduction in cytosolic movement in the *s4h* mutant compared to other genotypes suggests less plasmodesmata-mediated transport. It is counterintuitive that there was not more cytosolic movement observed in *s4h* compared to the wild type; since the *s4h* plasmodesmata look like type I, a high SEL would have been expected which might have allowed for increased eYFP transport (Nicolas et al. 2017; Gombos et al. 2023; Wegner and Ehlers 2024).

We do not expect that the rare movement of both cytosolic eYFP and ER luminal SP-mCer-KDEL in *s4h* reflects incomplete or disrupted cytokinesis, as inferred in *ipcst.92*. In *ipcst.92,* diffusion of both fluorophores was observed more frequently, and in significantly larger cell clusters (Fig. 5, G and H, Supplementary Fig. S5, A to C). Rather, we expect this motility in *s4h* is the result of either (1) two parallel bombardments of two adjacent cells, (2) a single microcarrier having penetrating two adjacent cells, or (3) bombardment of a cell actively undergoing cytokinesis. We expect that explanation two is especially likely with microcarriers impacting multiple *s4h* phyllid cells, due to the observed loss of regular anticlinal divisions in this genotype (Fig. 2, O to S).

### Modified plasmodesmal structure in phyllids of GIPC-deficient mutants does not abolish their general capacity for intercellular cytosolic transport

We observed strongly reduced transport of eYFP molecules in the *s4h* mutant, so the question arose whether these plasmodesmata are functional at all. This question was also provoked by the structural abnormalities of all mutant plasmodesmata observed by TEM. To test whether smaller molecules can move from cell to cell, we performed FRAP experiments on phyllids stained with the small fluorescent dye carboxyfluorescein diacetate (CFDA, broken down to and sequestered as carboxyfluorescein [CF], 376 Da, upon cellular uptake). We compared CF FRAP in parenchyma cells from phyllids dissected from six-week old gametophore tips. In all genotypes, we could find cells that did not recover fluorescence, even 10 or 20 min after photobleaching, and cells that partially or fully recovered fluorescence more or less quickly within this observation period (Supplementary Fig. S6, A to D). A true quantitative, kinetic comparison among genotypes is not possible due to the strong developmental phenotypes of the mutants. However, focusing on any potential differences specific to *s4h* (12 experiments), we observed similar frequency of no, partial, or complete CF redistribution within 10 min compared to wild type (10 experiments), and the redistribution occurred with similar kinetics (Supplementary Fig. S6, E to G). Redistribution of CF after bleaching in *s4h* demonstrates that despite its overall developmental phenotype (Figs. 1 to 3), strong plasmodesmal structural defects (Fig. 4) and less macromolecular transport (Fig. 5), the plasmodesmata likely retain some general functionality.

For the *ipcst.92* mutant it is impossible to eliminate the possibility that CF redistribution is affected by incomplete cell walls. However, we could identify cells which were less likely to be cytoplasmically connected to adjacent cells due to incomplete cell division by pre-examining their cell walls in Z-stacks of the dye-stained phyllids (Supplementary Fig. S6J). We noticed that cells with incomplete cell walls had a characteristic response to photobleaching: local loss of fluorescence was never complete, and recovered rapidly within a minute after bleaching (Supplementary Fig. S6K). With this approach the incomplete cell walls and intercellular motility of cytosol and endomembrane were even more obvious (also see Supplementary Video SV1). As observed previously with light and electron microscopy of thin-sectioned phyllids (Figs. 2, L and N; 3, A and B), here we could confirm that anticlinal cell wall gaps and attachment sites showed no consistent orientation or placement relative to the whole phyllid.

## Discussion

### Cell division and membrane morphology are impacted when GIPC synthesis is manipulated in *P. patens*

We isolated *P. patens* mutants with GIPC deficiencies including weak and strong overall reductions, and dramatic shifts in composition. One of the most severe deficiencies we observed in overall GIPC content in *P. patens* was in the *ipcs* triple mutant allele *ipcst.92*, which shows especially strong reductions in GIPCs glycosylated with hexose and pentose sugars, but no increase in free ceramide levels. We previously reported gross morphological defects in this mutant, and here we show that this dwarfism is coincident with a drastic reduction in cell size and especially cell number per phyllid. We infer a reduced frequency of cell division. Further analysis with light and electron microscopy linked this phenotype to incomplete anticlinal cell divisions. The incomplete cell walls show a surprisingly normal ultrastructure including the presence of plasmodesmata, suggesting that their occurrence is most likely not linked to general problems in the synthesis of cell wall material. This contrasts to the recently-reported impact of glycosylated sphingolipids (GlcCers/HexCers) on cellulose synthase complex velocity and cell wall polysaccharide content (Villalobos et al. 2024). Cellular bombardment and FRAP assays demonstrated that the openings within anticlinal cell walls allow for free movement of cytosol and ER between adjacent cells. In light of this dramatic ultrastructural defect, it is remarkable that the *ipcst.92* mutant is not only viable, but also produces differentiated cells: it can establish protonema and gametophores, including phyllids with hadroms, albeit with weaker structural definition than in the wild type. Notably, regeneration from gametophores to protonema in *ipcst.92* is slow; beginning with homogenization of gametophores, the production of homogenous protonema requires several months of weekly cultivation.

Observation of altered cell division associated with sphingolipid deficiency is not without precedent. Previous work described Fumonisin B1 (FB1)-treated *A. thaliana* roots, which are impaired in the synthesis of VLCFA-containing ceramides that normally channel into the assembly of GIPCs (Molino et al. 2014). FB1-treated plants exhibit complex shifts in their sphingolipid profiles, however two of the more substantial shifts are: (1) an overall increase in total free ceramide levels, and (2) a redistribution of the chain length profile of the fatty acid moieties of sphingolipids, namely an increase of ceramides with 16 carbon LCFAs that could partially compensate for diminished VLCFA-containing ceramides (Markham et al. 2011). FB1 treatment was linked to a mild reduction in cell size, and a more substantial ∼25% reduction in cell number, within the root division zone in *A. thaliana* (Molino et al. 2014). At present, we cannot distinguish whether the cytokinesis defects in *ipcst.92* are directly due to a reduction in GIPCs, or if they could be mediated by downstream effects on the overall fatty acid and lipid content of cellular membranes. Unfortunately, there are limited options for confirming the chemotype-to-developmental phenotype relationship in this mutant with pharmacological approaches. The *ipcst.92* mutant allele could not be phenocopied by treating *P. patens* with the IPCS inhibitor Aureobasidin A (AbA) (Supplementary Fig. S7, A to D). This resistance is not likely due to low AbA uptake by the moss; *PpIPCS2*, the most strongly-expressed *IPCS* in *P. patens* gametophores and protonema, imparts AbA resistance to host cells upon heterologous expression in *Saccharomyces cerevisiae*, which is otherwise susceptible to AbA due to inhibition of the endogenous IPCS AUR1 (AUREOBASIDIN A RESISTANT 1) (Supplementary Fig. S7, E to H).

A mechanism whereby GIPCs, as VLCFA-containing sphingolipids, could affect cytokinesis was previously proposed (Molino et al. 2014): VLCFAs could stabilize highly-curved membranes that form upon vesicle fusion, which is likely of particular importance during cytokinesis due to the volume of Golgi-derived vesicles fusing at the expanding cell plate. In both our *P. patens ipcst.92* mutant and in FB1-treated *A. thaliana* roots, plate formation appears to be aborted during late cytokinesis, after substantial vesicle fusion must have already occurred. If our model is correct, this implies there is sufficient VLCFA and/or GIPC content in these membranes to support the initial stages of vesicle fusion, but that at some point it can no longer support stable expansion of the cell plate. This hypothesis recalls observations in *S. cerevisiae* that VLCFA-containing lipids are essential for formation of the highly-curved membranes of nuclear pores (Schneiter et al. 2004).

Similar phenotypes to *ipcst.92* were also observed in *A. thaliana pi4kβ1 pi4kβ2* double mutants deficient in PI4P synthesis, that is, aborted cytokinesis producing multinucleate cells (Lin et al. 2019). The similarity of *P. patens ipcst.92* to *A. thaliana pi4kβ1 pi4kβ2* specifically is perhaps surprising in light of recent analysis of *Atipcs1/2* amiRNA lines, which revealed PIP phospholipase activity is dependent on the presence of VLCFA-containing sphingolipids in *A. thaliana* (Ito et al. 2021). This study demonstrated that reduced levels of VLCFA-containing sphingolipids produced increased PI4P at the TGN, that is, an inverse relationship between GIPC and PI4P accumulation. The similarity of our GIPC-deficient phenotypes to PI4P-deficient phenotypes is in apparent opposition with the notion that GIPC accumulation is antagonistic to PI4P accumulation. However, we did not measure phosphoinositide levels in *P. patens* for the present study, and our approach for GIPC detection is insensitive to subcellular distribution. More precise analysis of the spatiotemporal changes in sphingolipid and phosphoinositide content may present a more harmonious model of how these lipid classes interact. Additionally, it is not inconceivable that there may be differences between *A. thaliana* and *P. patens* in this respect, considering substantial differences between cytokinesis in these two models, reviewed in (Buschmann and Zachgo 2016; Buschmann and Müller 2019), and the fact that the tracheophyte and bryophyte lineages diverged over 450 million years ago.

The plasma membrane detachment phenotypes we observed in all mutants could support the notion that GIPCs impact the physical connection of the plasma membrane and cell wall. These phenotypes could also, alternatively or additionally, be driven by accumulation of excess plasma membrane material in the mutants, such that the plasma membrane area exceeds the inner cell wall area. Vesicular trafficking is likely impacted by GIPC deficiencies, given GIPC enrichment along the secretory pathway and the fact that GIPCs impact membrane physical properties: they increase membrane thickness due to their incorporation of VLCFAs (Levine et al. 2000), and increase lipid ordering due to their incorporation of saturated, hydroxylated LCB and VLCFA moieties that hydrogen bond with each other and free sterols in the membrane (Grosjean et al. 2015). Further, as discussed above, VLCFA-containing GIPCs are expected to be required for vesicle fusion during cytokinesis. It seems reasonable to expect that other dynamic processes involving plasma membrane budding and fusion would be impacted by sphingolipid content, including secretion and endocytosis, and this in turn would affect the staining properties and physical appearance of the plasma membrane (see summary of mutant phenotypes, Supplementary Fig. S8). Our observation of large, stellate vesicles in *ipcst.92*, and the tight, near-angular curvatures of plasma membrane in *s4h* could both be related to modified membrane rigidity.

### Aggregates in *s4h* phyllid cells resemble papillae formed at sites of pathogen ingress

The *s4h* mutant has a complex and unique metabolic phenotype: *s4h* accumulates sphingolipids with 18:0;2 LCBs in place of 18:0;3 LCBs, and has strong accumulations of B-series Hex-Hex-GIPCs and Hex-HexNAc-GIPCs. Among the many visual phenotypes observed in *s4h*, we were especially interested in the loss of orientation of cell division within phyllids. This observation together with the overall morphological abnormalities of the *s4h* mutant suggest that it may have partially lost the capability to define cell and organ polarity. Another interesting aspect of the *s4h* phenotype is the tufted aggregates observed here and in previous studies (Gömann et al. 2021; Steinberger et al. 2021). Based on distinct colors and staining properties observed by light microscopy, there appear to be two types of aggregates representing different chemical compositions. However, TEM analyses revealed only one type of aggregate, appearing to be composed of materials with distinct electron densities in a layered arrangement. The aggregates likely include callose, as suggested by aniline blue-stained accumulations adjacent to cell walls seen with epifluorescence microscopy, and electron-lucent layers within the depositions attached to cell walls in TEM images. Callose is involved in biotic and abiotic stress responses, and is a component of papillae that form at sites of attempted pathogen ingress (An et al. 2006). Besides the electron-lucent putative callose, the aggregates contain layers of electron-dense material with strong osmium tetroxide staining, which we speculate could be phenolic substances. Perhaps with increasing amounts these electron-dense components could incrust, harden, and impart some accumulations with the red color observed by light microscopy, and concomitantly mask the accessibility of the callose matrix for consistent aniline blue staining. Patchy callose fluorescence (Supplementary Fig. S3B) after aniline blue staining of the aggregates may indicate intermediate stages in this process. Further investigations on the chemical composition of the aggregates are required to test this hypothesis. Regardless, with the clues presently available, we speculate that the apparent co-deposition of callose and phenolics is not dissimilar from papillae. The available descriptions of papillae formed in mosses upon pathogen infection are strikingly similar to our observations in *s4h* (Davey et al. 2009; de León 2011; Yan et al. 2018). Plant responses to pathogens are strongly affected by sphingolipids, by multiple mechanisms (reviewed in Huby et al. 2020), and papilla formation at sites of attempted fungal ingress were recently specifically associated with GIPC metabolism in *A. thaliana* (Mittendorf et al. 2025). Therefore, in the *s4h* mutant, disruption of sphingolipid homeostasis could trigger a constitutive defence response. This was previously suggested by (Steinberger et al. 2021). Here, we can add that these structurally complex, papillae-like features are a compelling indication that this is the case. We noticed other signs of cell death in *s4h* that may reflect a constitutive hypersensitive response: fragmentation of chloroplasts was frequently observed in *s4h* with basic light microscopy (Supplementary Fig. S3E), as well as patchy CF fluorescence, suggesting inconsistent hydrolysis of non-fluorescent CFDA to produce fluorescent CF (Supplementary Fig. S6, H and I).

### Plasmodesmal development is impacted by GIPC deficiencies in *P. patens*

The *s4h* and *ipcst.92* mutant plasmodesmata did not undergo a full type I-to-type II(-like) transition (Nicolas et al. 2017; Wegner and Ehlers 2024). Additionally, we observed approximately two-fold higher increased plasmodesmal density in *s4h*, and four- to five-fold increased plasmodesmal densities in *ipcs2.1.11* and *ipcst.92* compared to the wild type. It could be that increased plasmodesmal density helps compensate for any modification to transport caused by plasmodesmal structural defects in these mutants. However, a model that could account for both the plasmodesmal structure and density phenotypes is that these reflect that the mutant phyllids are altogether not fully differentiated (Figs. 1 and 2). As such the phyllid cells of these mutants could be expected to display a default state, incomplete gametophore-specific programming, and/or may share some resemblance to protonema. Fittingly, *P. patens* protonemal cell walls are characterized by a very high density of type I plasmodesmata, and lack type II(-like) plasmodesmata (Gombos et al. 2023). A second model to explain both the changes in plasmodesmal density and structure is that the cytokinetic defects of *ipcst.92* and *s4h* cause their plasmodesmal phenotypes: It has been demonstrated that primary plasmodesmal deposition is determined by ER strands becoming trapped and integrated in the expanding cell plate (Hepler 1982; Li et al. 2024); perhaps the cytokinetic defects are somehow responsible for the higher frequency of ER “trapping” in the plate, and/or the stabilization and retention of desmotubules. Overall, it seems most likely that the plasmodesmal phenotypes observed in these sphingolipid-deficient mutants are at least partially secondary effects of broader developmental and/or cytokinetic defects. By converse reasoning, it is possible that the impact of modified plasmodesmal structure and density on intercellular transport should contribute to the gross morphological phenotypes of the mutants.

Some intercellular exchange of eYFP (27 kDa) was observed in all mutants, but was strongly reduced in *s4h*. A comparison can again be made to different *P. patens* developmental stages. Protonema exclusively form straight plasmodesmata at very high density (Gombos et al. 2023), and movement of Dendra2 (26.1 kDa) could be observed in protonema within 15 min. Between mature phyllid cells, which are connected by type II-like plasmodesmata (Wegner and Ehlers 2024 ), movement was only observed after 9.5 h over 1 to 2 cells (Kitagawa and Fujita 2015). The slow spread of Dendra2 into neighboring phyllid cells indicates transport of larger molecules may be restricted; however, it is not entirely prevented by a strict size exclusion limit (Kitagawa and Fujita 2015). This raises the question of whether the SEL is indeed an exclusively qualitative and definite parameter; it could be that it reflects random leakage that is unavoidable during targeted macromolecule transport. The reduced transport in *s4h*, with its type I-like plasmodesmata, further underlines the fact that the type I-to-type II transition cannot be used to determine SEL. Thus, we infer that developmental control of the plasmodesmal SEL is exerted in regions of the plasmodesmata largely unaffected by the transition from type I to type II and instead presumably occurs via molecular changes in the neck regions at the plasmodesmal orifices. Callose deposition at plasmodesmata is also known to affect plasmodesmal permeability by restricting the diameter of the neck regions (Schreiber et al. 2024 ; Tee and Faulkner 2024), including in bryophytes (Renzaglia et al. 2024; Hsu et al. 2025). Recent findings have suggested that sphingolipid modulation controls the proper localization of enzymes involved in callose turnover (Iswanto et al. 2024). Yet, we have not found any indication that plasmodesmal callose levels significantly affected the SEL in our GIPC-deficient mutants, as mature *s4h* plasmodesmata showed the smallest callose deposits of the mutants despite allowing for the least eYFP transport. In contrast, compared to wild-type plasmodesmata, inner neck diameters were significantly reduced in all three mutants; this was the only structural feature consistent with the observed reduced macromolecular transport rate in *s4h*.

In *A. thaliana*, multiple C2-domains and transmembrane region proteins (MCTPs) have been shown to act as plasmodesmata-specific ER-plasma membrane tethers that interact with PI4P, and contribute to plasmodesmal formation and function (Li et al. 2024; Pérez-Sancho et al. 2025). *A. thaliana* MCTP-deficient mutants also show dwarfism and aberrant cell division phenotypes (Brault et al. 2019) that are similar to *P. patens s4h*. MCTPs were identified in the *P. patens* plasmodesmal proteome, and confirmed to localize to plasmodesmata via confocal microscopy (Gombos et al. 2023). It will certainly be of interest to functionally characterize MCTPs in *P. patens* and to test whether their localization, and the localization of other plasmodesmal and plasma membrane proteins are disrupted in mutants such as *s4h*. MCTP function, rather than callose deposition, may be more directly linked to the control of macromolecular transport between cells (Pérez-Sancho et al. 2025).

## Materials and methods

### Cultivation

*P. patens* ecotype Gransden 2004 was obtained from the International Moss Stock Center (IMSC; https://www.moss-stock-center.org/en/), strain 40001. Plants were maintained under long-day conditions (16 h light/8 h dark, at 25 °C/18 °C) with 105 to 120 *μ*mol m^−2^ s^−1^ radiation. Filamentous protonema tissue was cultivated on BCD-AT medium (1 mm MgSO_4_, 1.84 mm KH_2_PO_4_, 10 mm KNO_3_, 45 *μ*M FeSO_4_, 5 mm ammonium tartrate, 1 mm CaCl_2_, Hoagland's trace elements, 0.55% plant agar) overlaid with sterile cellophane discs, and the cultures were propagated by disruption in sterile tap water with an IKA ULTRA-TURRAX homogenizer every 1 to 2 weeks. Development of gametophores was induced by transferring small pieces of tissue to BCD medium (BCD-AT lacking ammonium tartrate, “AT”). Gametophores were sub-cultivated every 4 to 8 weeks (Maronova and Kalyna 2016). The *s4h* mutant was generated *via* homologous recombination as described (Gömann et al. 2021), with the entire coding sequence replaced by a kanamycin selection marker. As the mutant lesions and phenotypes are indistinguishable in the two alleles described therein, no differentiation was made between these for the present work, aside from their presentation in Supplementary Fig. S2. Generation of the *ipcs* mutant series with CRISPR/Cas9 was previously reported (Haslam et al. 2024); *ipcs2.1.11.* contains a single nucleotide insertion 76 bp from the ATG start of *IPCS2*, resulting in an early frame shift in the coding sequence. The *ipcst.92* mutant has frame-shift lesions in *ipcs1* (16 bp deleted 32 bp downstream from the ATG start) and *ipcs3* (16 bp deletion and 2 bp substitution, 189 bp downstream from the ATG start), while the *ipcs2* lesion is an out-of-frame 12 bp deletion, resulting in the substitution of IELTV > M, a putative alternative translational start. Our previous work demonstrated via allele replacement in *P. patens* and heterologous expression in *S. cerevisiae* that this specific *ipcs2* lesion impairs but does not eliminate IPCS2 activity (Haslam et al. 2024).

### General growth phenotype analyses

Gametophore colony area and circularity were measured from eight week-old -plants. After ventilating plates under a sterile bench to reduce humidity and condensation, mutant and wild-type gametophores were photographed directly in covered petri dishes, using a Retiga R6 camera on an Olympus SZX12 stereomicroscope. Image analysis was carried out with ImageJ 1.53f51 (Wayne Rasband and contributors, National Institutes of Health, USA) (Schneider et al. 2012). Area was measured by selecting of gametophores with the Wand tool and adjusting the selection tolerance. Gametophore circularity was calculated as C = 4 πA/P^2^ (C = circularity; A = area; P = perimeter) (Vidali et al. 2007; Vidali and Bezanilla 2012).

Cell number per phyllid and cell size were measured from phyllids excised from mature gametophore colonies. Phyllids were imaged on an Olympus BX51 light microscope with a 10× objective, and a Retiga R6 camera. We selected expanded but non senescent phyllids, ideally #6 to 12 from the apex of each gametophore shoot for measurements. For counting total cell #/phyllid, we counted cells on one side of the hadrom and multiplied this number by two. Again, measurements were carried out manually with ImageJ 1.53f51.

Semi-thin sections of 0.5 to 1 *µ*m thickness were cut from the median plane of fixed and resin-embedded phyllids with glass knives on a Reichert Om U2 ultramicrotome (Leica Microsystems GmbH, Wetzlar, Germany) for histological investigations, using the same samples on which TEM was performed (see “Transmission electron microscopy” below) (Wegner and Ehlers 2024). Sections were stained with 0.5% crystal violet and observed under a Leica DM 5500 microscope B equipped with a Leica DFC 450 camera driven by the Leica Application Suite software (Version 4.3.0).

### Aniline blue staining and combined light and fluorescent microscopy

Live cell staining with aniline blue paired with light and epi-fluorescent microscopy was used to compare the localization and test for colocalization of different pigmented, stained, or fluorescent compounds observed in various studies of the *s4h* mutant. Live cell staining followed the protocol described by (Sankoh et al. 2024). Gametophore phyllids were then imaged on two separate Olympus BX51 light microscopes, one fitted with a Retiga R6 color camera, and another with a black and white Hamamatsu ORCA-Flash4.0 LT+ camera. An F46-001 ET-Set CFP filter cube was used for aniline blue and F41-007 HQ-Set Cy3 for autofluorescence.

### Biolistic transformation

Marker plasmids were bombarded into four-week-old gametophore colonies (or five- to eight-week-old for the additional, supplemental experiments in Supplementary Fig. S5). Within experiments, each different genotype (or one representative allele of each genotype) was cultivated on the same 5 cm Petri plate, and were therefore transformed with the same plasmid preparations, precipitated on the same microcarriers, and bombarded in the same shot. Free enhanced yellow fluorescence protein (eYFP, 27 kDa) was expressed from a pUC18-derived entry vector, driven by the 35S promoter. The ER marker plasmid was received from Prof. Ralf Reski, University of Freiburg; this consisted of the *P. patens* aspartic protease signal peptide fused to mCerulean (27 kDa) with C-terminal KDEL retention signal (SP-mCer-KDEL), driven by the *P. patens ACTIN5* promoter, in a pJET1.2-based vector (Mueller and Reski 2015).

Microcarriers were prepared and precipitated on macrocarriers as described (Müller et al. 2017), with minor modifications. Briefly, 60 mg of 1.0 *μ*m gold particles (Biorad, #1652263) were washed in 1 mL 70% ethanol, rinsed in ultra-pure water, and suspended in 1 mL 50% glycerol, for a final concentration of 60 mg/mL.

To prepare microcarriers sufficient for two bombardments, 4 *μ*g of each plasmid to be transformed or co-transformed was used to coat a 10 *μ*L aliquot of the 60 mg/mL gold suspension. The 10 *μ*L aliquot of gold was first vortexed, the plasmid DNA added, and then sterile water, to bring the mixture volume up to 55 *μ*L. Then 50 *μ*L 2.5 m CaCl_2_ and 20 *μ*L 0.1 m spermidine were added. The mixture was vortexed for 2 min then briefly spun down at low speed. The carriers were washed twice in 96% ethanol, and finally re-suspended in 60 *μ*L 96% ethanol, then 2 × 30 *μ*L were dispersed on macrocarriers (Biorad, #1652335) for bombardment. Gametophores were bombarded in a Bio-Rad PDS-1000/He Biolistic Particle Delivery System, with 900 psi rupture discs, and the vacuum pressure raised to 24 inHg before bombardment. After bombardment, gametophores were cultivated under normal growth conditions for two to three days before imaging.

### Symplastic motility assays

Images of bombarded cells from expanded phyllids approximately # 6 to 12 from the gametophore apex were captured on a Zeiss Axio Imager.Z2 LSM980 confocal laser scanning microscope, with a Plan-Apochromat 20×/0.8 M27 objective. eYFP was excited with a 514 nm diode laser with a 26 *μ*m/0.9 AU pinhole, with emission detected between 525 and 569 nm. Chlorophyll and SP-mCer-KDEL were both excited with a 405 nm diode laser with a 26 *μ*m/1.0 AU pinhole; chlorophyll fluorescence was detected between 645 and 713 nm, and SP-mCer-KDEL between 460 and 525 nm. Images were processed with ZEN3.2 and ImageJ 1.53f51. Lower-magnification images of bombarded cells were also captured on an Olympus BX51 microscope with eYFP detected using an F41-028 HQ filter set, with a Hamamatsu ORCA-Flash4.0 LT+ camera. For assessing the direction of fluorophore movement, we reasoned that all cell types present in mature *P. patens* phyllids are longer in the proximal-distal axis and shorter across the width of the phyllid. Therefore, movement across the short cell walls was used as a proxy for proximal-distal movement, and movement across the long cell wall as a proxy for lateral movement.

### Carboxyfluorescein fluorescence redistribution (/recovery) after photobleaching (FRAP)

Whole, 6-week-old *P. patens* gametophores were stained in aqueous 1 mm 5-(and-6)-carboxyfluorescein diacetate (CFDA, fluorescent and membrane impermeant upon hydrolysis to carboxyfluorescein, CF, 376 Da) for 60 to 90 min at RT, and subsequently washed four times in water. The gametophores were incubated in tap water overnight in the dark at room temperature to improve stain distribution. During this long incubation period, some CF was sequestered from the cytoplasm into the vacuoles.

FRAP was carried out with a Zeiss Axio Imager.Z2 LSM980 confocal laser scanning microscope, with a Plan-Apochromat 40×/1.4 oil immersion objective. CF was bleached 50× at 2.52 s/frame with 100% intensity of 405 and 488 nm diode lasers. The pinhole was 28 *μ*m/0.88 AU. CF fluorescence was detected with the 488 nm laser at 1 to 3% intensity, between 507 and 537 nm with a multi-alkali photomultiplier tube (PMT). Images were analyzed with ZEN3.2 and processed with Image J 1.53f51 (Wayne Rasband and contributors, National Institutes of Health, USA).

### Transmission electron microscopy

Sample preparation for light and electron microscopy followed the protocol of (Wegner and Ehlers 2024). Whole gametophores were embedded in 2% (w/v) low gelling agarose (type VII Sigma-Aldrich, Steinheim, Germany) and solidified agarose blocks were fixed in 50 mm phosphate buffer containing 2.5% (v/v) glutaraldehyde (GA) at pH 7.23 for 2 h at RT and for another 2 h on ice. After rinsing in 100 mm buffer, postfixation was performed in 0.9% (w/v) OsO_4_ in 0.1 m buffer at 4 °C overnight. Rinsing in water was followed by staining for 2 h with 0.5% (w/v) aqueous uranyl acetate on ice, and dehydration in a graded ethanol series and propylene oxide. Samples were embedded in Spurr's epoxy resin (adapted mixture; (Spurr 1969)) and polymerized at 68 °C for 20 h.

Ultrathin sections (∼80 nm) were cut with a diamond knife, transferred onto formvar-coated single-slot copper grids, and contrasted with 2% uranyl acetate and lead citrate (Reynolds 1963) for 12 min each. Sections were analyzed with an EM912AB TEM (Zeiss, Oberkochen, Germany) at 120 kV accelerating voltage under zero-loss energy filtering conditions. Micrographs were recorded with a 2k × 2k dual-speed slow-scan CCD camera (SharpEye, TRS, Moorenweis, Germany) using the iTEM software package (OSIS). Figure plates were mounted with Corel PHOTO-PAINT (2021, Version 23.1.0.389, Corel, Ottawa, Canada) and in Adobe Illustrator CS6 Version 16.0.3, measurements (including plasmodesmal neck diameters) were performed with ImageJ 1.53e (Wayne Rasband and contributors, National Institutes of Health, USA) (Schneider et al. 2012). For plasmodesmal densities, we applied the strict criterion to count only those plasmodesmal strands which could unambiguously be identified under the TEM, and clearly extended into the electron-opaque cell wall layers. For calculations, we used the equation described by (Gunning 1978), following the method described in (Wegner and Ehlers 2024).

### Lipidomic analysis

Gametophores were harvested and lyophilized after four or eight weeks of growth, depending on the growth rate of the genotype.

#### Microsome enrichments

Microsome enrichments followed a published protocol (Abas and Luschnig 2010). 10 mg of lyophilized tissue were taken from each sample, and ground with stainless steel beads in a laboratory mixer mill MM400 (Retsch GmbH, Haan, Germany). The pulverized tissues were then suspended in a fractionation buffer consisting of 150 mm Tris-HCl (pH 7.5), 37.5% sucrose, 7.5% glycerol, 15 mm EDTA, 15 mm EGTA, and 7.5 mm KCl. The suspensions were vigorously mixed, then centrifuged at 1,500 g for 3 min at 4 °C. The supernatant was retained, and the pellet re-extracted twice with the fractionation buffer diluted first 0.75 X, then 0.67 X. All of the supernatants were pooled, diluted 1:1 with water, then spun at approx. 21,000 g for 2 hr. The pellets were washed, resuspended in water, re-spun for 45 min, then finally sealed under argon gas, snap frozen in liquid nitrogen, and stored at −80 °C.

#### Lipid extractions

Total lipid extraction followed a published protocol (Herrfurth et al. 2021). Microsome pellets were resuspended in 6 mL extraction buffer consisting of isopropanol:hexane:water in a 60:26:14 ratio, v:v:v. They were vortexed, sonicated, and shaken at 60 °C for 30 min. Cell debris was then spun down at 800 g for 20 min at room temperature. The supernatant was dried under a nitrogen stream and finally resuspended in 800 *μ*L tetrahydrofuran:methanol:water in a 4:4:1 ratio, v:v:v (TMW). The samples were stored at −20 °C, or processed directly.

#### Methylamine treatment

Methylamine treatment followed the protocols of (Markham and Jaworski 2007; Herrfurth et al. 2021). Lipid extracts used for sphingolipid measurement were treated with methylamine to hydrolyze glycerolipids. This fraction was dried under a nitrogen stream, then reuspended in 700 *μ*L 33% methylamine in ethanol (v/v) and 300 *μ*L water. The samples were incubated at 50 °C for 1 h, and then dried again under nitrogen. The residue was finally resuspended in TMW.

#### Ultra-high pressure liquid chromatography coupled with nanoelectrospray and tandem mass spectrometry (UPLC-nanoESI-MS/MS)

Sample injection of 2 *µ*L was performed using an autosampler set at 18 °C and sample separation was conducted at a flow rate of 0.1 mL/min. The solvent system was composed of methanol:20 mm ammonium acetate (3:7, v/v) with 0.1% acetic acid (v/v) (solvent A) and tetrahydrofuran:methanol:20 mm ammonium acetate (6:3:1, v/v/v) with 0.1% acetic acid (v/v) (solvent B). A linear gradient was applied: start from 65% B for 2 min; increase to 100% B in 8 min; hold for 2 min and re-equilibrate to the initial conditions in 4 min. For multiple reaction monitoring (MRM), precursor ions were [M + H]^+^ and product ions were dehydrated LCB fragments for ceramides and GlcCers. The loss of phosphoinositol-containing head groups was used for the detection of GIPCs.

#### Fatty acid methyl ester (FAME) derivatization and measurement by gas chromatography with flame ionization detection (GC-FID)

20% of the total lipid extract was used for methylesterification to FAMEs, and total fatty acid quantification for normalizing the peak areas of individual lipid species measured by high performance UPLC-nanoESI-MS/MS. Aliquots were dried under nitrogen gas, and resuspended in a sulfuric acid solution (267 mL MeOH, 133 mL toluene, 11 mL sulfuric acid, 8 mL dimethoxypropane, based on (Garcés and Mancha 1993)). 5 *μ*g tripentadecanoin standard was added to each sample, and the derivatization was carried out for 1 h in an 80 °C water bath. The reaction was stopped by the addition of 200 *μ*L of saturated, aqueous NaCl, and the FAMEs extracted in hexane. The final extract was dried under nitrogen, and re-suspended in acetonitrile for injection into an Agilent 6890N GC-FID.

### Accession numbers

Sequence data from this article can be found in the GenBank/EMBL data libraries under accession numbers (gene version 3.3/6): *IPCS1*, Pp3c8_19970V3.1/Pp6c8_10430V6.1; *IPCS2*, Pp3c23_1080V3.1/Pp6c23_510V6.1, *IPCS3*, Pp3c20_10120V3.1/Pp6c20_6540V6.1; *S4H*, Pp3c23_17650V3.1/Pp6c23_8710V6.1.

## Supplementary Material

kiaf549_Supplementary_Data

## Data Availability

The data underlying this article are available in the article, in its online supplementary material, and upon request to the corresponding author.

## Supplementary data

The following materials are available in the online version of this article.

**Supplementary Figure S1**. Sphingolipid content of mutant gametophores.

**Supplementary Figure S2**. Gross morphological phenotypes of mutant gametophores.

**Supplementary Figure S3**. Abnormal aniline blue-stained accumulations occur in gametophores of the *s4h* mutant.

**Supplementary Figure S4**. Comparative transmission electron microscopy of organelles and other cell compartments observed in healthy phyllid parenchyma cells.

**Supplementary Figure S5**. Intercellular connectivity is consistently affected in gametophores of *ipcst.92* and *s4h*, while phenotypes are not observed in *ipcst.92* and *s4h* protonema.

**Supplementary Figure S6**. FRAP of cytosolic carboxyfluorescein in phyllids of wild-type and GIPC-deficient mutants over time.

**Supplementary Figure S7**. *P. patens* IPCS2 is resistant to the mycotoxin IPCS inhibitor Aureobasidin A (AbA).

**Supplementary Figure S8**. Broad summary of mutant phenotypes.

**Supplementary Video SV1**. Representative fluorescence redistribution after photobleaching experiment of an *ipcst.92* phyllid cell.

## Dive Curated Terms

The following phenotypic, genotypic, and functional terms are of significance to the work described in this paper:

inositol CHEBI: CHEBI:24848

ceramide CHEBI: CHEBI:17761

sphinganine-C4-hydroxylase Gramene: Pp3c23_17650V3.1
sphinganine-C4-hydroxylase Araport: Pp3c23_17650V3.1

inositol phosphate ceramide synthase1 Gramene: Pp3c8_19970V3.1
inositol phosphate ceramide synthase1 Araport: Pp3c8_19970V3.1

aureobasidin A resistant1 Gramene: AUR1/YKL004W
aureobasidin A resistant1 Araport: AUR1/YKL004W

inositol phosphate ceramide synthase2 Gramene: Pp3c23_1080V3.1
inositol phosphate ceramide synthase2 Araport: Pp3c23_1080V3.1

inositol phosphate ceramide synthase3 Gramene: Pp3c20_10120V3.1
inositol phosphate ceramide synthase3 Araport: Pp3c20_10120V3.1
