# Supplementary material for: Complex sphingolipid metabolism impacts cell division and plasmodesmal development in the moss Physcomitrium patens
Source: Plant Physiol. 2025 Nov 5;199(3):kiaf549. doi: 10.1093/plphys/kiaf549 (PMC12619088; doi:10.1093/plphys/kiaf549)
Supplement: kiaf549_Supplementary_Data [file kiaf549_supplementary_data.zip › Supplementary Figures.pdf]

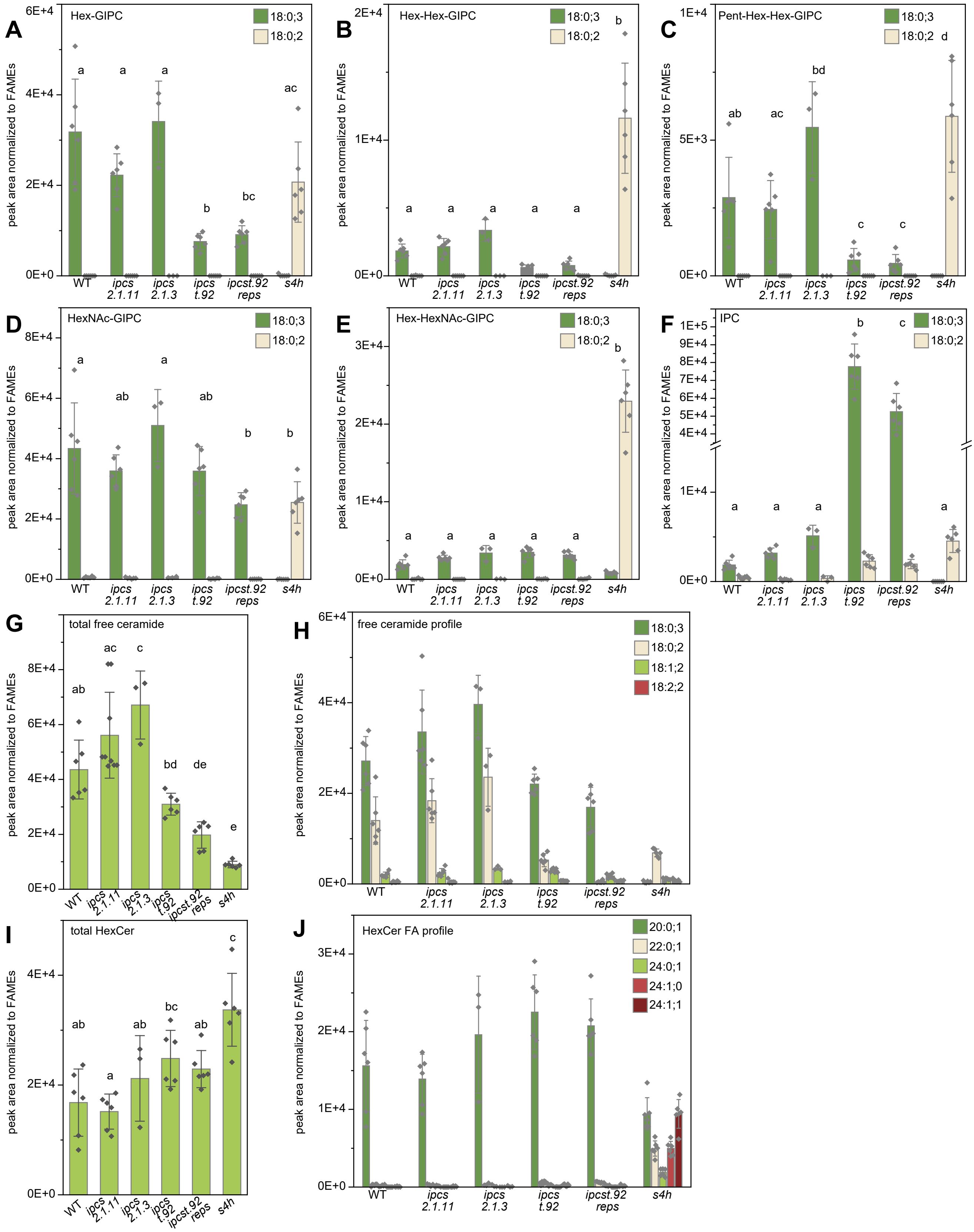

Supplementary Figure S1: Sphingolipid content of mutant gametophores. (**A-F**) GIPC profiles by headgroup, with measurements classified by LCB moiety (18:0;3 and 18:0;2). (**G**) Total free ceramides, and (**H**) free ceramide profile classified by LCB moiety. (**I**) Total HexCer, and (**J**) HexCer profile classified by FA moiety (20:0;1, 22:0;1, 24:0;1, 24:1;0, 24:1;1). Measurements are UPLC-nanoESI-MS/MS quantification of lipids extracted from microsome enrichments from gametophores. Amounts are individual or summed peak areas of MRMs, normalized to the fatty acid content in each sample determined by FAMEs analysis. Additional mutant alleles (*ipcs2.1.3* and *ipcst.92* replicas) were included to verify phenotype consistency. Plotted values are averages of three or six biological replicates, bars represent standard deviation. Letters indicate significance at $p < .05$ determined by one-way ANOVA with Tukey's *post-hoc* test.

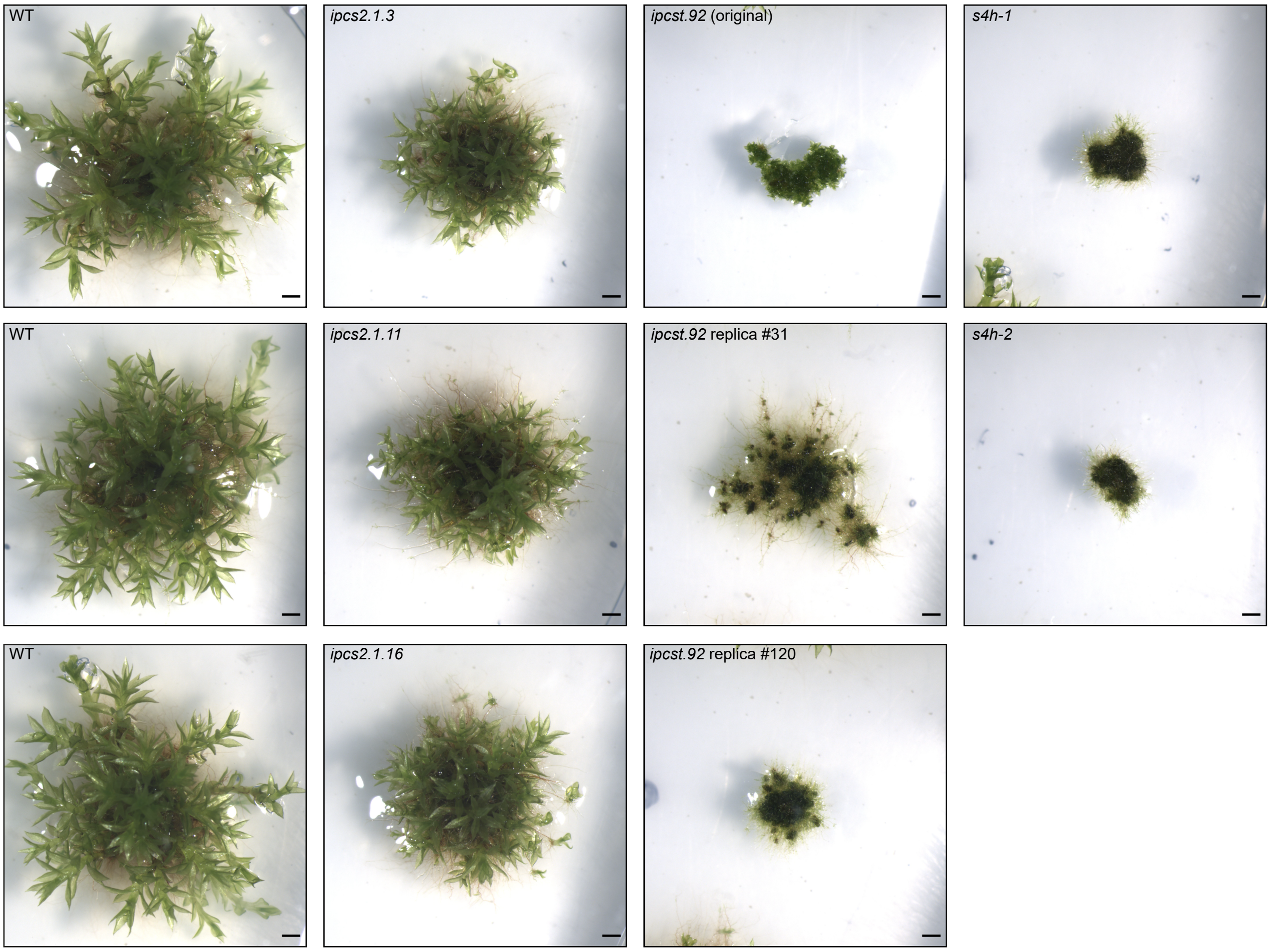

Supplementary Figure S2: Gross morphological phenotypes of mutant gametophores. All plants were grown on the same BCD media plate for six weeks under standard growth conditions, as described in the Methods. Images representative of three replicates, grown on different plates. All scale bars represent 1 mm.

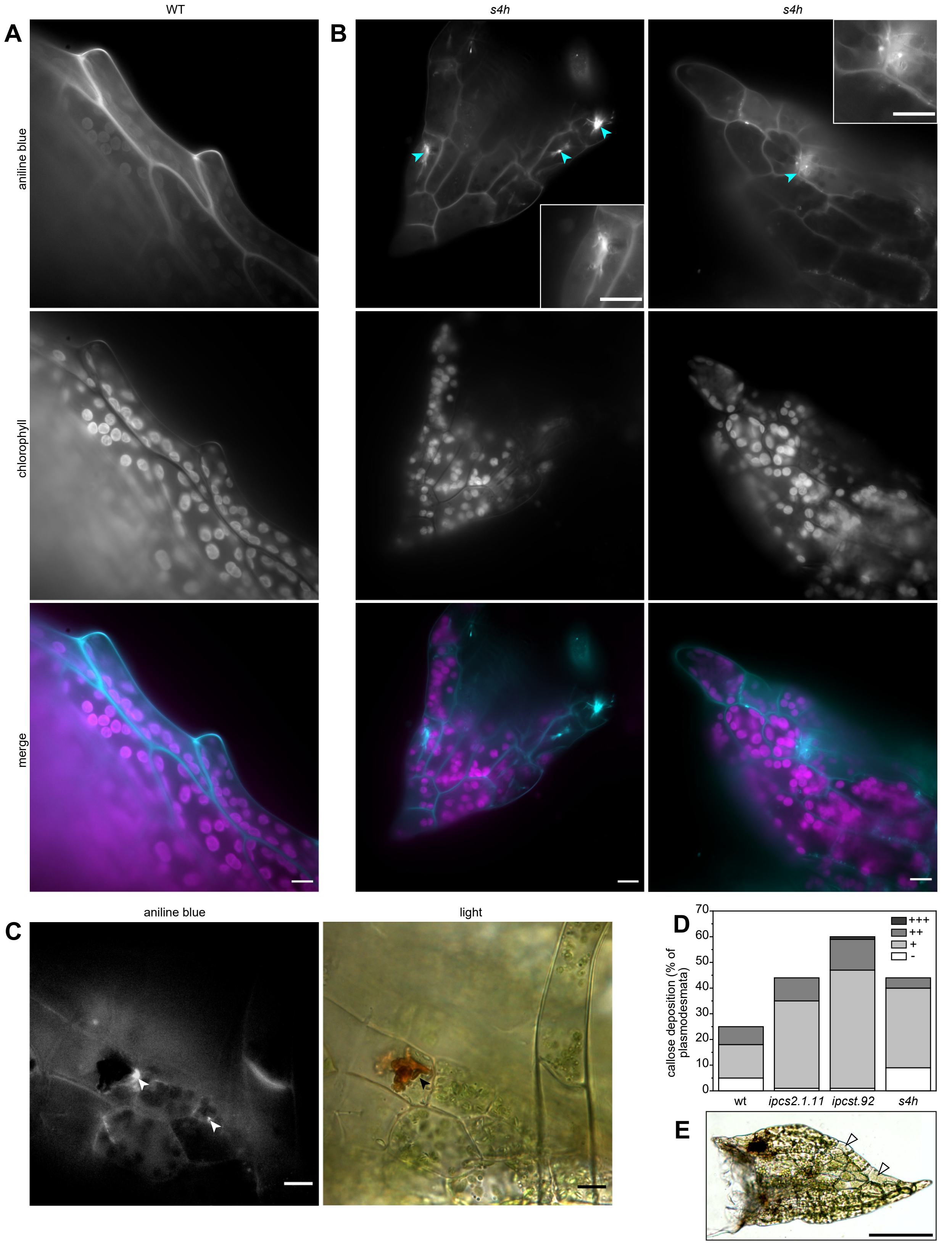

Supplementary Figure S3: Abnormal aniline blue stained accumulations occur in gametophores of the *s4h* mutant. (**A**) Wild-type phyllids are stained with aniline blue primarily in cell walls dividing adjacent cells, while staining is less intense in cell walls facing the environment, and absent within cells. (**B**) *s4h* phyllids are also stained in cell walls dividing adjacent cells, and additionally show patches of bright intracellular staining (turquoise arrowheads). Magnified insets emphasize patchy aniline blue staining (**C**) Aniline blue stained accumulations in *s4h* phyllids (white arrowheads) do not co-localize with red-pigmented deposits (black arrowhead) that also accumulate in phyllids of this mutant. Note that monochrome images (**A**,**B**) were captured with a different microscope and camera than the color image (**C**). Scale bars are 10 µm. (**D**) Assessment of callose deposition at plasmodesmal neck regions observed by TEM. Callose deposits were classified into four categories (+++ very large amounts; ++: large amounts; +: moderate amounts; and -: no/minor amounts of callose). Percentages of plasmodesmata whose callose deposits fall into the respective categories are shown for each genotype. Only those plasmodesmata whose necks were sectioned in a median plane were taken into account. The data provide the basis for the depiction of callose deposits in the schematic drawings of plasmodesmata in Figure **4V**. (**E**) Representative image of an *s4h* phyllid showing not only the presence of red deposits, but also chloroplast fragmentation (open arrowheads). Sacle bar is 0.1 mm.

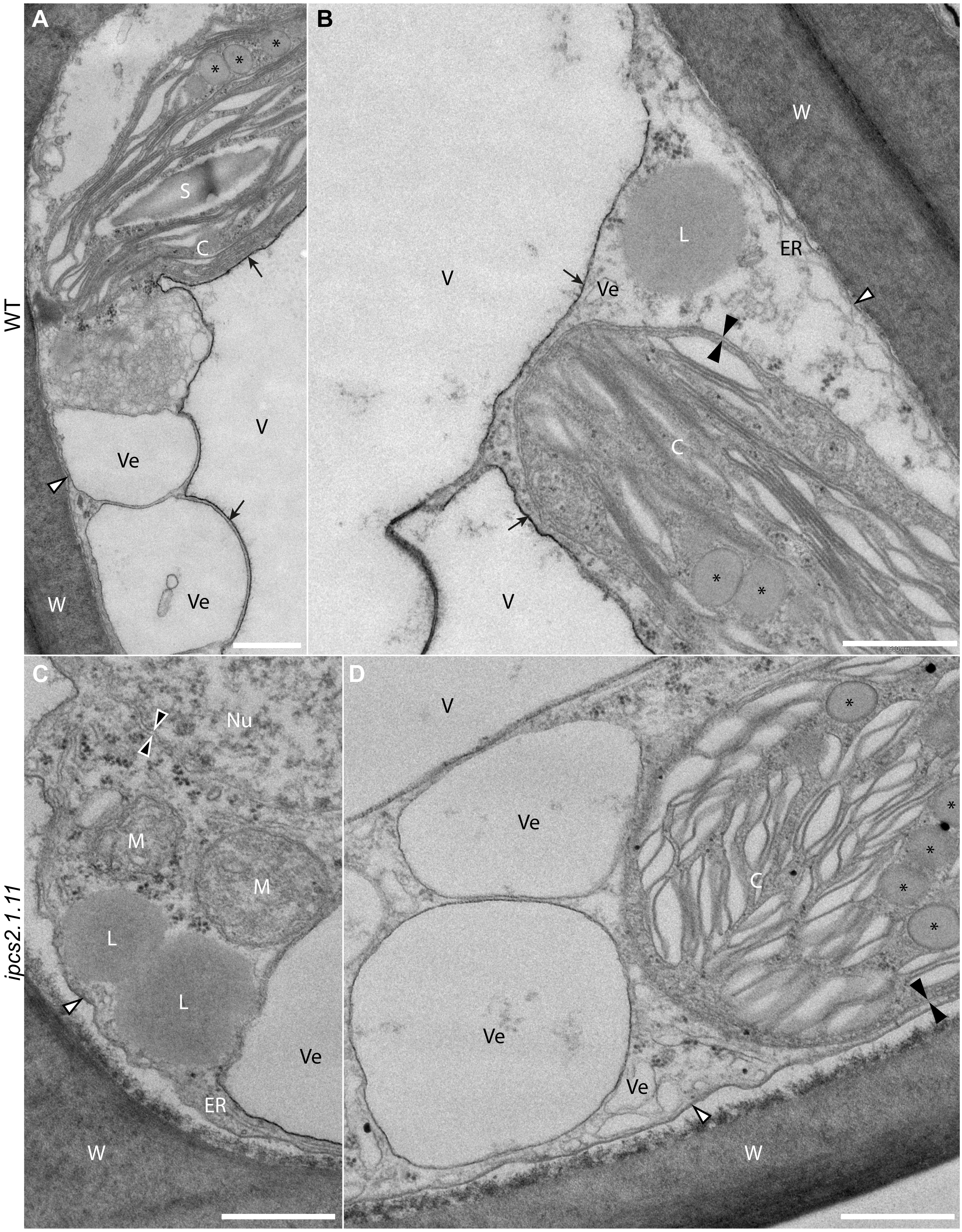

Supplementary Figure S4: Comparative transmission electron microscopy of organelles and other cell compartments observed in healthy phyllid parenchyma cells. (**A**-**B**) Wild type, (**C**-**D**) *ipcs2.1.11* single mutant, (**E**-**G**) *ipcst.92* triple mutant, and (**H**-**L**) *s4h* mutant. Beyond the main mutant phenotypes described in Fig. 3, the overall ultrastructure of the *ipcs* and *s4h* mutant cells largely resembled that of the wild type, with the following exceptions. Larger vesicles in the *ipcst.92* cells often had an irregular stellate shape (**E**) compared to the other genotypes, perhaps related to changes to membrane rigidity. In the *s4h* mutant, the mitochondrial matrix was often more electron lucent (**I, J**) than in the other genotypes (**C, F, G**). The images also provide further evidence for the peculiarities of the plasma membrane-cell wall adherence of the *ipcs2.1.11* (**C**,**D**), *ipcst.92* (**F**), and *s4h* (**H**) mutant cells, described in Fig. 3. C: chloroplast; ER: endoplasmic reticulum; L: lipid body; M: mitochondrion; Nu: nucleus; S: starch grain; V: vacuole; Ve: vesicle; W cell wall; asterisks: plastoglobuli; black arrows: strongly stained tonoplast; black arrowheads: chloroplast envelope; black arrowheads with white margins: nuclear envelope; white arrowheads with black margins (/open arrowheads): plasma membrane; white arrows: mitochondrial envelope. Scale bars: 500 nm.

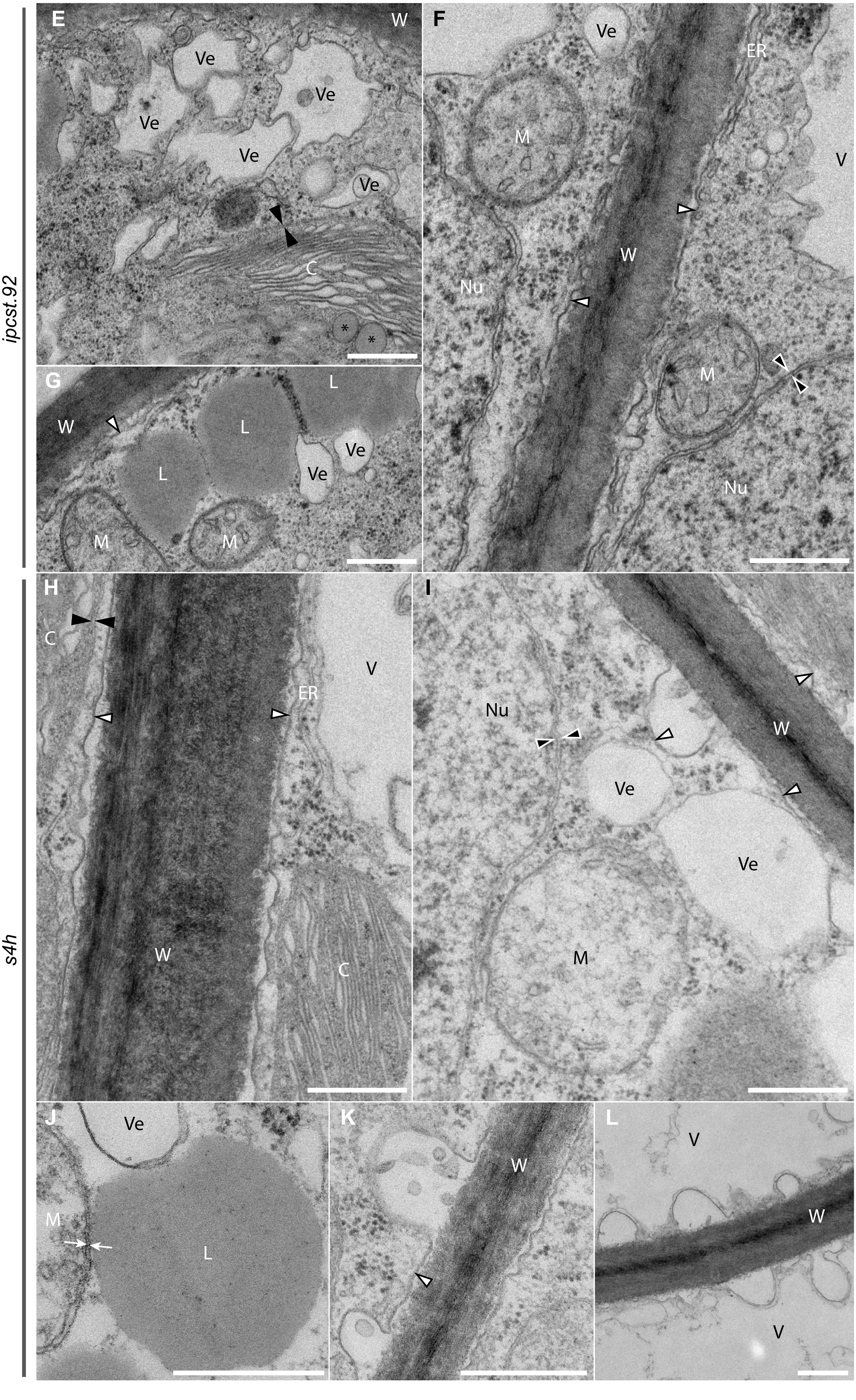
ipcst.92
s4h
E
W
Ve
Ve
Ve
Ve
Ve
C
F
Ve
ER
M
V
W
Nu
M
Nu
G
L
W
L
Ve
L
Ve
M
M
H
C
V
ER
W
C
I
Nu
W
Ve
Ve
M
J
Ve
M
L
K
W
L
V
W
V

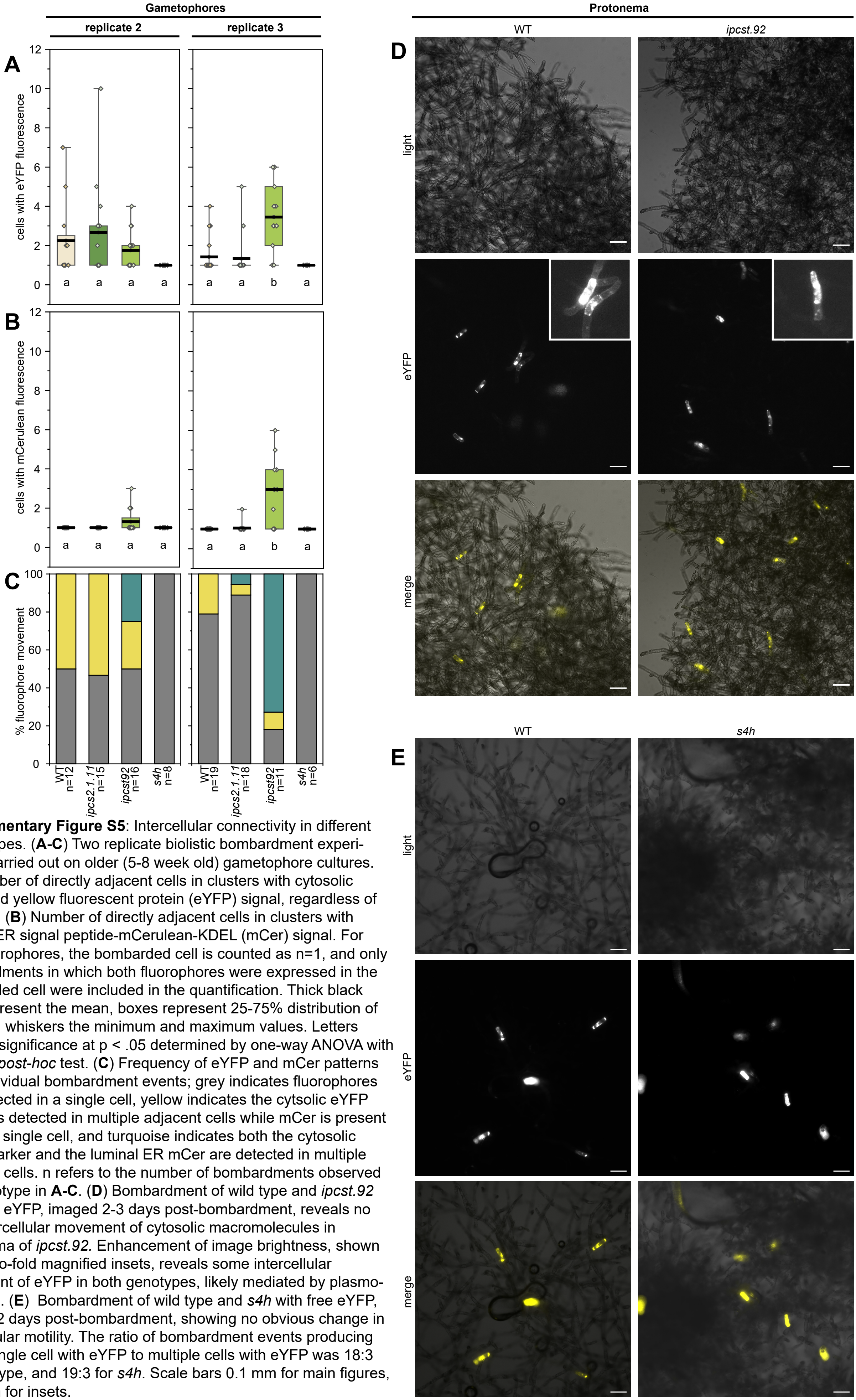

Supplementary Figure S5: Intercellular connectivity in different tissue types. (**A-C**) Two replicate biolistic bombardment experiments carried out on older (5-8 week old) gametophore cultures. (**A**) Number of directly adjacent cells in clusters with cytosolic enhanced yellow fluorescent protein (eYFP) signal, regardless of intensity. (**B**) Number of directly adjacent cells in clusters with luminal ER signal peptide-mCerulean-KDEL (mCer) signal. For both fluorophores, the bombarded cell is counted as n=1, and only bombardments in which both fluorophores were expressed in the bombarded cell were included in the quantification. Thick black lines represent the mean, boxes represent 25-75% distribution of the data, whiskers the minimum and maximum values. Letters indicate significance at $p < .05$ determined by one-way ANOVA with Tukey's *post-hoc* test. (**C**) Frequency of eYFP and mCer patterns after individual bombardment events; grey indicates fluorophores only detected in a single cell, yellow indicates the cytsolic eYFP marker is detected in multiple adjacent cells while mCer is present in only a single cell, and turquoise indicates both the cytosolic eYFP marker and the luminal ER mCer are detected in multiple adjacent cells. n refers to the number of bombardments observed per genotype in **A-C**. (**D**) Bombardment of wild type and *ipcst.92* with free eYFP, imaged 2-3 days post-bombardment, reveals no free intercellular movement of cytosolic macromolecules in protonema of *ipcst.92.* Enhancement of image brightness, shown in the two-fold magnified insets, reveals some intercellular movement of eYFP in both genotypes, likely mediated by plasmodesmata. (**E**) Bombardment of wild type and *s4h* with free eYFP, imaged 2 days post-bombardment, showing no obvious change in intercellular motility. The ratio of bombardment events producing only a single cell with eYFP to multiple cells with eYFP was 18:3 for wild type, and 19:3 for *s4h*. Scale bars 0.1 mm for main figures, 0.05 mm for insets.

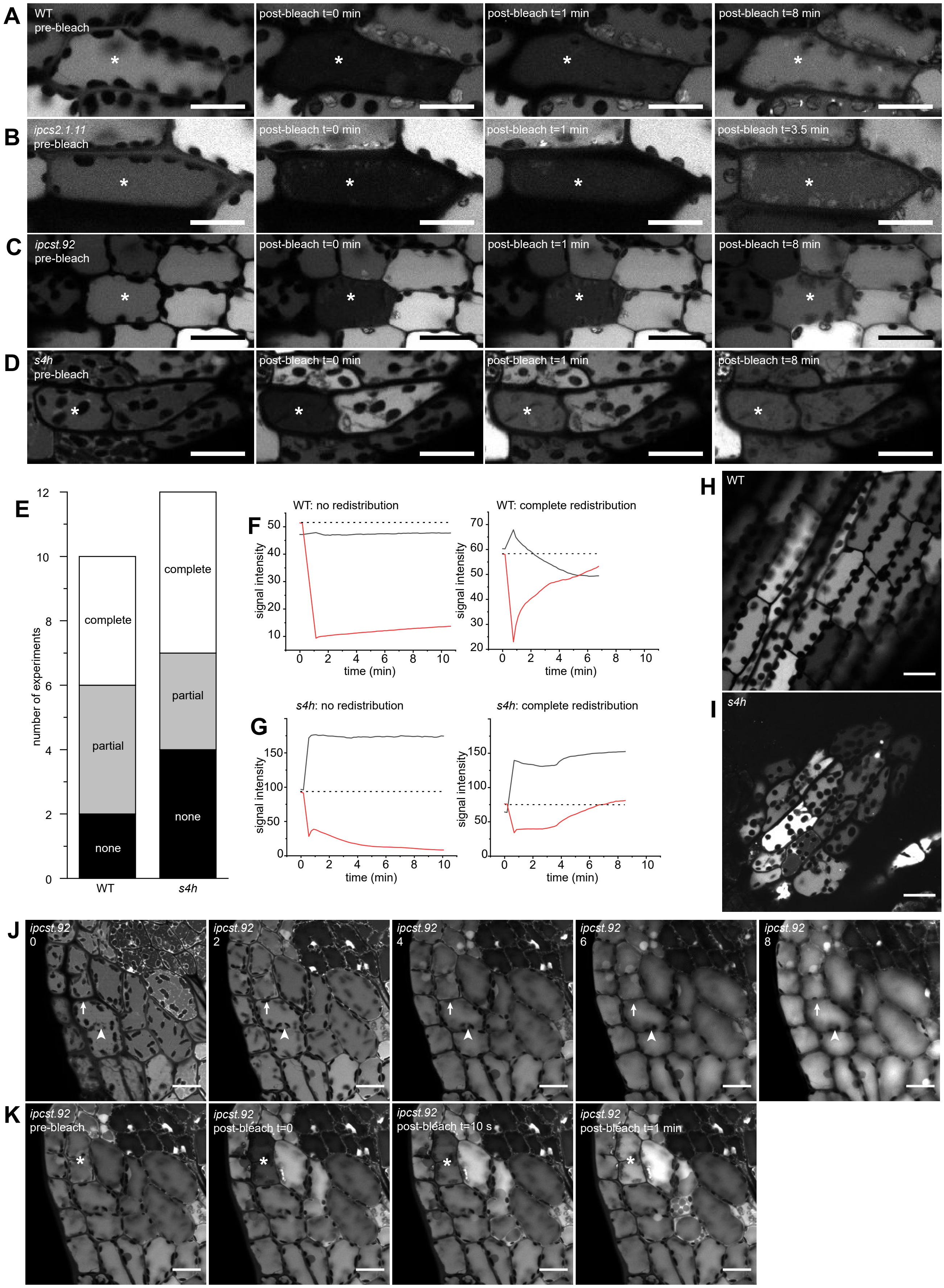

Supplementary Figure S6: FRAP of cytosolic carboxyfluorescein in phyllids of wild type and GIPC-deficient mutants over time (t). A Wild type **B** *ipcs2.1.11* **C** *ipcst.92* **D** *s4h*. Asterisks mark the bleached cells. **E** Frequency of complete and partial fluorescence redistribution in wild type and *s4h*. Only experiments in which the target cell was completely bleached and where there were no obvious signs of stress were included in the totals. The ratio is not indicative of a greater or lesser tendency to fluorescence recovery, but rather the capacity for some degree of intercellular CF transport in these two genotypes. **F-G** Representative fluorescence intensity curves from wild type and *s4h*, extracted from selected experiments represented in **E** showing either no redistribution or complete redistribution. Red lines are the signals from bleached cells, dark grey lines are the signals from control cells, which are similar in size and shape to bleached cells and located nearby but not in direct contact with the bleached cell. The dashed lines represent the initial signal intensity of the bleached cell. Experiments were initially set up for 10 min, but were stopped if the signal had fully recovered or plateaued. **H-I** Representative image of consistent CF fluorescence in wild type (**H**) and patchy CF fluorescence in *s4h* (**I**) as also observed in *ipcst.92*. **J-K** Incomplete cell divisions in the *ipcst.92* mutant are identifiable after application of CFDA, in Z-stacks and fluorescence redistribution after photobleaching (FRAP). (**J**) Representative images from a Z-stack series, images shown here were captured 2 µm apart. The white arrow indicates a cell wall that appears complete at the top of the stack, and incomplete at the bottom. The cell above this cell wall was bleached in (**K**). The white arrowhead indicates a cell wall that, conversely, is absent at the top of the stack, and complete at the bottom. (**K**) FRAP of a cell with an incomplete cell wall, the bleached cell is marked with an asterisk. All scale bars are 20 µm.

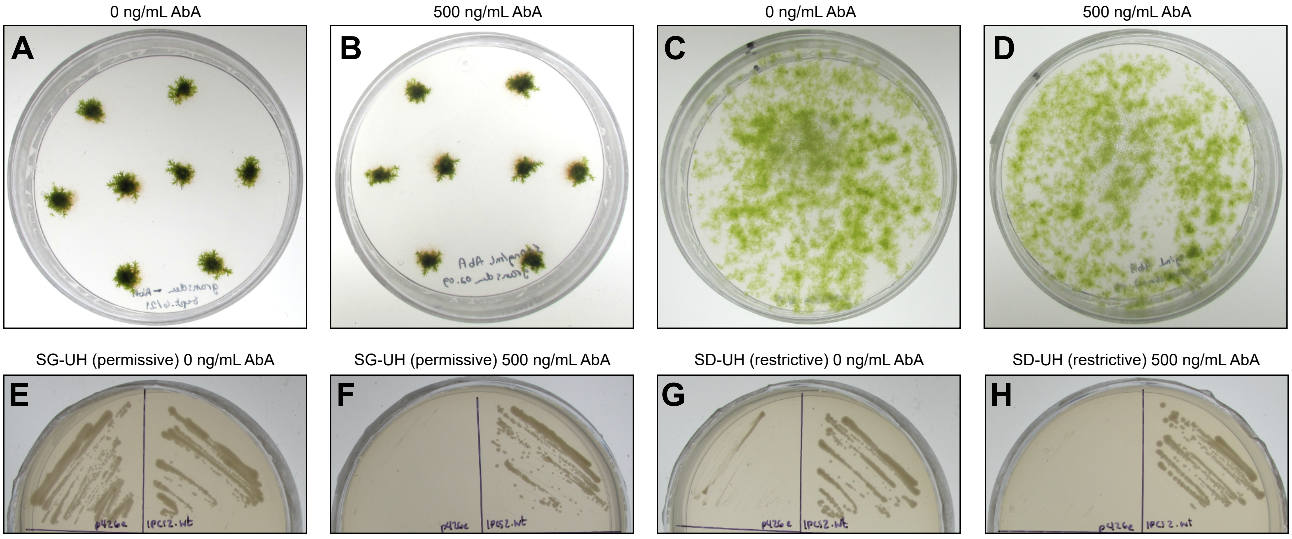

Supplementary Figure S7: *P. patens* IPCS2 is resistant to the mycotoxin IPCS inhibitor Aureobasidin A (AbA). (**A-D**) AbA treatment does not impact growth and development. (**A-B**) Gametophores grown for 35 days (**A**) without and (**B**) with 500 ng/mL AbA. (**C-D**) Protonema grown for 11 days (**C**) without and (**D**) with 500 ng/mL AbA. (**E-H**) *PpIPCS2* expression is sufficient to rescue the AbA susceptibility of *Saccharomyces cerevisiae*, caused by the inhibition of the endogenous ScIPCS, AUREOBASIDIN A RESISTANT (AUR1). *PpIPCS2* was expressed in the mutant line YPH499-HIS-GAL1:AUR1, in which the endogenous ScAUR1 promoter is replaced by the galactose-inducible promoter, GAL1. Both in conditions where the endogenous ScAUR1 is induced (SG, **E** and **F**) and suppressed (SD, **G** and **H**), PpIPCS2 can sustain growth of the yeast on 500 ng/mL AbA (**F** and **G**).

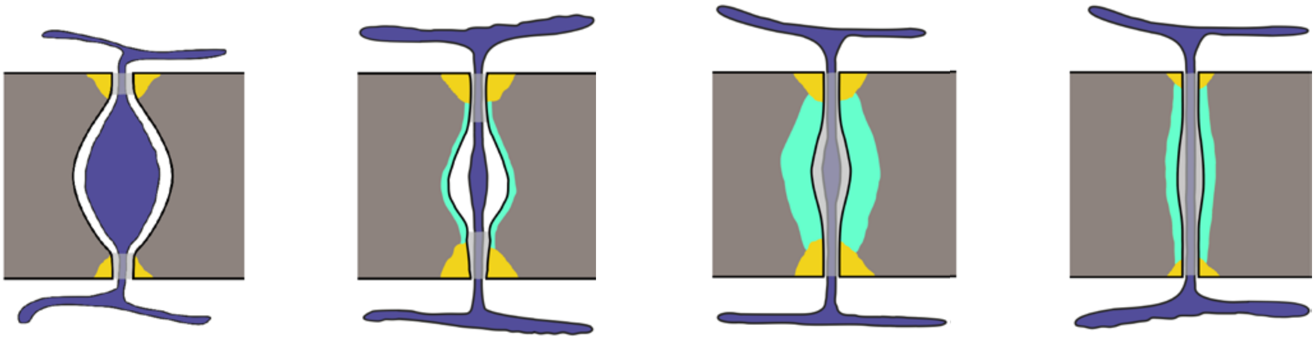

Genotype	*ipcs2.1.11*	*ipcst.92*	*s4h*
Chemotype	• Weakly depleted A-series Hex-GIPCs	• Strongly reduced A,B,C-series Hex-GIPCs<br>• Strongly accumulating IPCs	• Strongly accumulating B and C series GIPCs<br>• Reduced free ceramides, modified free and complex ceramide profiles<br>• Weak accumulation of HexCer
Morphology	• Reduced colony area<br>• Reduced phyllid length	• Strongly reduced colony area<br>• Increased circularity<br>• Strongly reduced phyllid length	• Strongly reduced colony area<br>• Increased circularity<br>• Strongly reduced phyllid length
Anatomy and histology	• Reduced overall cell number/phyllid<br>• Slight reduction in margin/edge cell length	• Dramatically reduced cell number, size, and impaired differentiation<br>• Hadrom cells fewer, wider, and with thinner cell walls<br>• Blunt-ended incomplete cell walls, producing irregularly wide cells	• Dramatically reduced cell number, size, and impaired differentiation<br>• Poorly-discernable hadrom without obvious stereids<br>• Irregular cell division planes, producing multilayered phyllid sections<br>• Cell wall aggregates with layered substructure and hard consistency
Cytological features	• Broad detachments of the plasma membrane from cell wall<br>• Fringed inner cell wall layers	• Rippled plasma membrane with frequent, small invaginations detached from the cell wall<br>• Large stellate vesicles	• Frequent, deep plasma membrane invaginations with high electron density<br>• Mitochondrial matrix less electron dense
Plasmodesmal structure	• Density increased 4X<br>• Type II (dilated PM and constricted ER, whereas in WT both dilated)<br>• Median dilations less prominent and shorter<br>• Often constricted desmotubules<br>• Regularly, moderately-sized cell wall collars<br>• Narrower necks	• Density increased 5X<br>• Mostly type I (constricted) with dense interior<br>• Median dilations rare, when present less prominent and short<br>• Often constricted desmotubules<br>• Often, moderate to strong cell wall collars<br>• Narrower necks	• Density increased 2X<br>• Mostly type I (constricted) with dense interior<br>• Median dilations rare, when present less prominent and short<br>• Often constricted desmotubules<br>• Frequent, moderate cell wall collars<br>• Narrower necks
Plasmodesmal function	• Similar to wild type	• Difficult to determine due to interference of incomplete cell division	• Reduced transport of macromolecules (eYFP, 27 kDa)

Supplementary Figure S8: Summary of mutant phenotypes, all relative statements in comparison to Gransden wild type.
