# Supplementary material for: Complex sphingolipid metabolism impacts cell division and plasmodesmal development in the moss Physcomitrium patens
Source: Plant Physiol. 2025 Nov 5;199(3):kiaf549. doi: 10.1093/plphys/kiaf549 (PMC12619088; doi:10.1093/plphys/kiaf549)
Supplement: kiaf549_Supplementary_Data [file kiaf549_supplementary_data.zip › Supplementary Video Legends.docx]

**Supplementary Video Legends**

**Supplementary Video SV1:** Representative fluorescence redistribution after photobleaching experiment of an *ipcst.92* phyllid cell. The photobleached region is outlined in red, and a nearby region used as reference for changes in carboxyfluorescein fluorescence over time without bleaching is outlined in cyan. To demonstrate the dimensions of the continuous cell clusters which are connected by incomplete cell walls, a cluster is shown with blue arrows indicating the gaps in the wall.
